# The long-term effects of insulin use in incident cystic fibrosis-related diabetes: a target trial emulated using longitudinal national registry data

**DOI:** 10.1183/23120541.00170-2022

**Published:** 2022-11-07

**Authors:** Emily Granger, Ruth H. Keogh, Freddy Frost

**Affiliations:** 1 Dept of Medical Statistics, Faculty of Epidemiology and Population Health, London School of Hygiene and Tropical Medicine, London, UK; 2 Institute of Infection, Veterinary and Ecological Sciences, University of Liverpool, Liverpool, UK

## Abstract

**Introduction:**

Cystic fibrosis-related diabetes (CFRD) is a common complication of cystic fibrosis and is associated with deleterious clinical outcomes. Insulin is recommended as a treatment by international guidelines. However, there are scarce clinical trial data to support the use of insulin, and little is known about the long-term outcomes of treatment. The aim of this study was to compare the long-term impacts of insulin use *versus* non-use in CFRD.

**Methods:**

We used data from the national UK Cystic Fibrosis Registry and adopted a target trial framework. Eligible individuals included those 12 years and older with a new diagnosis of CFRD. Outcomes were change in % predicted forced expiratory volume in 1 s (FEV_1_ %) and body mass index z-scores (BMI) over a 5-year follow-up period. Treatment strategies were to receive insulin or not for the duration of follow-up. Treatment effect estimates were obtained using two methods to control for confounding: inverse-probability-of-treatment weighted estimation of marginal structural models and the G-formula.

**Results:**

We identified 1613 individuals diagnosed with CFRD between 2008 and 2016 and included 1196 and 1192 in the FEV_1_ % and BMI outcome analyses respectively. We found no evidence of an effect of insulin on FEV_1_ % over the 5-year study period. Similarly, we found no overall effect of insulin on BMI; however, there was some evidence for a positive treatment effect in patients with lower baseline BMI.

**Conclusion:**

Using well-established national registry data, we found no evidence of long-term treatment effects for insulin on FEV_1_ % or BMI in people with incident CFRD.

## Abstract 2

**This target trial using registry data to estimate the effects of insulin use on clinical outcomes in incident cystic fibrosis-related diabetes found no evidence of a long-term benefit (up to 5 years) of insulin use on lung function or BMI** https://bit.ly/3B8azKz

## Introduction

Cystic fibrosis (CF) is an autosomal recessive disease characterised by thickened epithelial secretions resulting in multi-system disease and end-organ damage. While progressive respiratory failure is the most common cause of death, CF-related diabetes (CFRD) is one of the most common extrapulmonary manifestations of the disease and has been found to be associated with poorer respiratory function, increased microbiological pathogen acquisition and increased mortality [1–3]. The pathophysiology of CFRD is complex and distinct from other forms of diabetes, but insulin deficiency secondary to damaged islets is the primary mechanism, with insulin resistance and incretin axis abnormalities also implicated to a lesser and more variable extent [4–6].

Current national and international guidelines recommend insulin as the only treatment for CFRD in children, adolescents and adults, yet the evidence supporting these recommendations is limited [1, 7, 8]. For example, while multiple small, uncontrolled, observational studies have described improvements in weight and/or lung function following insulin initiation in people with CFRD [9–11], there have been few prospective clinical trials [12, 13]. A recent Cochrane systematic review identified just four studies suitable for inclusion, and only one measured outcomes beyond 12 months [12, 14]. The benefits observed in small observational studies have not been replicated in clinical trials, and the Cochrane review found no evidence of a benefit for insulin over oral hypoglycaemic agents [14].

According to UK Cystic Fibrosis (CF) Registry data, 89% of people receiving treatment for CFRD are prescribed insulin, and the corresponding figure from the US CF Registry is 81% [15, 16], which equates to approximately a third of all adults with CF in each country receiving insulin.

Given the widespread utilisation of insulin in CFRD, understanding the long-term outcomes associated with treatment is of paramount importance. Routinely collected healthcare databases or well-established national patient registries that contain data on treatment use, health outcomes and other key covariates provide the opportunity to study treatment effects over long time periods, when coupled with appropriate analytical methods to account for confounding by indication. National registries are well established in CF but have not previously been harnessed to study treatment effects in CFRD. Given the lack of long-term clinical trial data, the aim of this study was to evaluate the long-term effects of insulin use in CFRD on lung function and body mass index (BMI) z-scores for up to 5 years from CFRD diagnosis using data from the UK CF Registry.

## Methods

### Study design and data source

We used data from the UK CF Registry, a national database managed by the Cystic Fibrosis Trust (NHS ethics approval reference: 07/Q0104/2). The registry has previously been described in detail [17]. Data are collected in a standardised way at approximately annual visits and have been recorded in a centralised database since 2007. At each annual review visit it is recorded whether the individual was diagnosed with CFRD since the previous visit. In the UK, CFRD can be diagnosed using continuous glucose monitoring, oral glucose tolerance test or serial glucose testing [7]. HbA1c-based diagnosis is not recommended due to high red blood cell turnover in CF, reducing the sensitivity of HbA1c to detect dysglycaemia [1, 7, 8].

For individuals diagnosed with CFRD, at each annual visit it is recorded whether they have, over the past year, been treated with insulin, oral hypoglycaemic agents, recommended dietary changes or no treatment. We considered two outcomes: lung function measured using % predicted forced expiratory volume in 1 s (FEV_1_ %) and BMI z-score. FEV_1_ % was calculated using the Global Lung Initiative equations [18]. BMI z-scores were calculated using the World Health Organization (WHO) reference distribution [19].

We also make use of data on the following covariates: sex, genotype, age, use of pancreatic enzyme supplements, use of nutritional support (including enteral and parenteral nutrition) and presence of *Pseudomonas aeruginosa* or *Burkholderia cenocepacia* complex infection. These covariates are recorded annually, except for sex and genotype.

### The target trial

Our study was designed to emulate a hypothetical randomised controlled trial (the “target trial”) comparing the effects of insulin use to no insulin use on FEV_1_ % and BMI in people with CFRD [20]. The target trial framework involves describing the protocol for the randomised trial one would like to conduct if it were feasible, and then emulating that target trial using the available observational data, and there are a growing number of epidemiological studies using this approach. The protocol for our target trial and the corresponding emulated trial are outlined in table 1. The key difference between the target trial and the emulated trial based on the observational data is that the analysis must account for the lack of randomisation, as far as possible. In the observational data, the association between insulin and outcomes is believed to be confounded by a number of factors, including time-varying factors. Furthermore, individuals switch between different treatments. The statistical analysis used to address these issues is outlined below.

**TABLE 1:** Protocol for the target trial investigating the impact of insulin use on cystic fibrosis-related diabetes (CFRD) outcomes and the corresponding emulated trial using UK Cystic Fibrosis Registry data

Protocol component	Target trial	Emulated trial
**Eligibility criteria**	Include: Individuals diagnosed with CFRD in the UK aged 12 years and older at time of diagnosis Exclude: Individuals who have had an organ transplant or are taking oral corticosteroids or CFTR modulators, prior to CFRD diagnosis	Include: Individuals observed in the UK CF Registry and labelled with CFRD between 2008 and 2016, meeting criteria in the target trial and who had data for at least one visit within 2 years prior to CFRD diagnosis and at least 1 year of follow-up after diagnosis Exclude: As in the target trial. We also exclude people with missing data on baseline confounders, including outcome at baseline, or missing data on infection or pancreatic insufficiency during the follow-up period
**Treatment strategies**	1) Initiate insulin at CFRD diagnosis and continue to take it throughout follow-up 2) Do not initiate insulin at CFRD diagnosis and continue not to take insulin throughout follow-up. Individuals in the no insulin group may use other non-insulin treatments for CFRD	As in the target trial
**Assignment procedures**	Participants will be randomly assigned to a treatment strategy when they are diagnosed with CFRD and will be aware of the strategy to which they have been assigned	In the emulated trial individuals are not randomly assigned to the treatment strategy, which is addressed in the analysis
**Follow-up period**	1, 2, 3, 4 and 5 years from diagnosis	As in the target trial
**Outcome**	We consider two outcomes: 1) FEV_1_ % (obtained using GLI equations) 2) Body mass index (BMI) z-score	As in the target trial
**Causal contrasts of interest**	Per-protocol	As in the target trial
**Analysis plan**	Mean difference in outcome between treatment groups at follow-up, adjusted for baseline level. Estimated using a linear regression model for the outcome, with treatment group and baseline measure of the outcome as explanatory variables	Confounding by measured baseline and time-varying covariates is addressed using IPTW of MSMs or G-formula (see section “Statistical analysis”)

CFRD: cystic fibrosis-related diabetes; CF: cystic fibrosis; CFTR: cystic fibrosis transmembrane conductance regulator; FEV_1_ %: % predicted forced expiratory volume in 1 s; GLI: Global Lung Function Initiative; BMI: body mass index; IPTW: inverse-probability-of-treatment weighting; MSMs: marginal structural models.

### Statistical analysis

The treatment effects of interest were the expected differences in outcomes at time horizons of 1–5 years had all individuals been given insulin on CFRD diagnosis (and continued to take insulin up to the time horizon of interest) *versus* had all individuals not been given insulin (and continued not to take insulin up to the time horizon of interest). Treatment effect estimates were obtained using two methods: inverse-probability-of-treatment weighting (IPTW) estimation of marginal structural models (MSMs) and the G-formula [21], which control for confounding in two different ways. Briefly, IPTW involves weighting subjects using time-dependent weights that are the inverse of the probability of them receiving the treatment history they received up to each time point conditional on their covariate history. A model for the outcome conditional on treatment history up to that time (the MSM) is then fitted using the weights. IPTW often produces extreme weights which can result in wider confidence intervals, and so stabilised weights were used to address this issue [22]. Alternatively, the G-formula uses a generalisation of standardisation to a longitudinal setting to estimate the same effects. This method requires models for the time-varying confounders at each time point conditional on the past history of treatment, confounders and outcomes. By considering two methods that require specification of different models, we assess the robustness of our findings to different assumptions. Some individuals were censored before 5 years of follow-up, either due to the administrative end of follow-up, death, organ transplant or initiating treatment with long-term corticosteroids or cystic fibrosis transmembrane conductance regulator (CFTR) modulators. This censoring was addressed using inverse probability of censoring weighting. Weights were also used to handle missing data on FEV_1_ % and BMI. Further details are provided in the supplementary material.

We used a directed acyclic graph to inform which variables needed to be accounted for (supplementary figure S1). Based on this, in the analyses of both outcomes, we controlled for confounding by the variables listed in section “Study design and data source” and additionally for measurements of FEV_1_ % and BMI z-score measured at the visit prior to CFRD diagnosis.

We considered MSMs for the outcome with and without interaction terms between treatment and the outcome measured at baseline. These were used to obtain treatment effect estimates conditional on people having specific values of FEV_1_ % and BMI z-score at baseline, representing high, moderate or low values. High, moderate and low FEV_1_ % was defined as values of 100, 75 and 40% predicted, respectively. High, moderate and low BMI z-score was defined as the 80th, 50th and 20th percentiles of the distribution of BMI z-scores at baseline. Wald tests comparing models with and without interaction terms were conducted to assess the evidence for an interaction.

Standard errors, 95% confidence intervals (CI) for effect estimates and the tests for interactions were estimated using the non-parametric bootstrap approach. Further details on the implementation of methods are provided in the supplementary material.

## Results

### Study population and descriptive statistics

**FIGURE 1:**
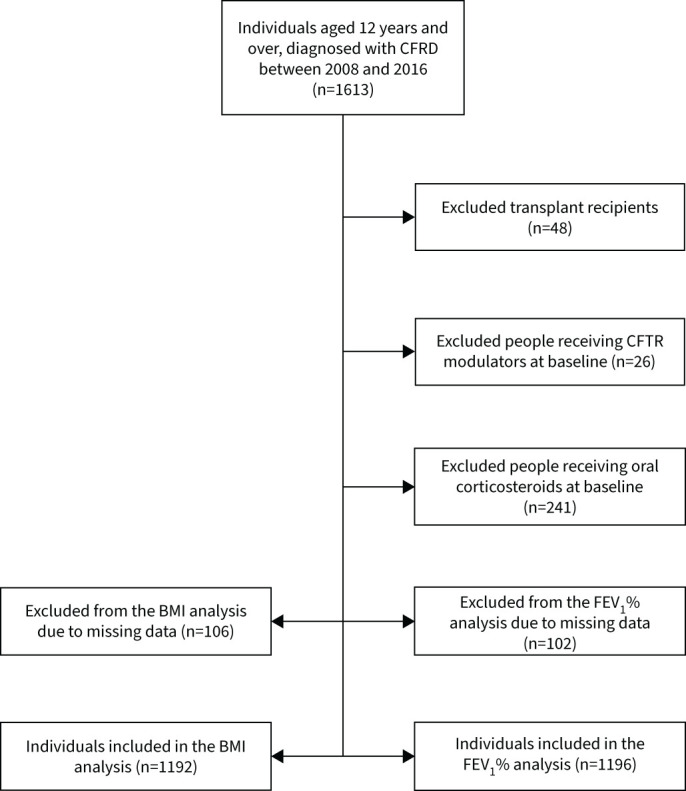
Flow chart of participant selection. CFRD: cystic fibrosis-related diabetes; CFTR: cystic fibrosis transmembrane conductance regulator; FEV_1_ %: % predicted forced expiratory volume in 1 s; BMI: body mass index.

1196 individuals met our criteria for inclusion in the emulated trial for the FEV_1_ % outcome, and 1192 met the criteria for the BMI z-score outcome (figure 1). Of the 1196 individuals in the FEV_1_ % analysis, 5-year follow-up was available for 634 (53.0%). 88 (7.4%) were censored due to death or transplant. There was a total 4404 patient-years of follow-up. Further details on the number of individuals censored by year in both the FEV_1_ % and BMI analyses are given in supplementary tables S1 and S2.

Table 2 summarises the characteristics (measured in the year prior to CFRD diagnosis), and number of follow-up years, of the 1196 individuals included in the FEV_1_ % analysis, by treatment status insulin use at baseline. Compared to those receiving insulin at baseline, those not receiving insulin at baseline were older (mean±sd age: 25.3±12.0 *versus* 22.1±9.0 years) and included fewer females (48.6% *versus* 51.4%). They had similar mean±sd FEV_1_ % (64.6±22.0% *versus* 64.2±21.4% and higher BMI z-score (−0.11±1.29 *versus* −0.28±1.20). During the follow-up period, the mean FEV_1_ % decreased by year, whereas the mean BMI z-score tended to increase by year (supplementary figure S4).

**TABLE 2:** Summary of characteristics at baseline by insulin use at baseline

	No insulin	Insulin
**Subjects n**	488	708
**Female**	237 (48.6)	364 (51.4)
**Age years**	25.3±12.0	21.9±9.0
**Genotype^#^**		
High risk	395 (80.9)	618 (87.3)
Low risk	33 (6.8)	14 (2.0)
Not assigned	60 (12.3)	76 (10.7)
**FEV_1_ %**	64.6±22.0	64.2±21.4
Change in previous 12 months^¶^	−1.0±10.6	−2.8±10.5
**BMI z-score**	−0.11±1.29	−0.28±1.20
Change in previous 12 months^¶^	−0.04±0.50	0.00±0.60
***Pseudomonas aeruginosa* infection**	295 (60.5)	468 (66.1)
***Burkholderia cenocepacia* complex infection**	28 (5.7)	28 (4.0)
**S*taphylococcus aureus* infection**	195 (40.0)	302 (42.7)
**Pancreatic enzyme supplements use**	418 (85.7)	637 (89.9)
**Maximum years of post-baseline follow-up**		
1	55 (11.3)	79 (11.2)
2	58 (11.9)	106 (15.0)
3	78 (16.0)	118 (16.7)
4	75 (15.4)	81 (11.4)
5	222 (45.5)	324 (45.8)

Continuous variables are summarised using mean±sd and categorical variables are summarised using n (%). FEV_1_ %: % predicted forced expiratory volume in 1 s; BMI: body mass index. ^#^: genotype risk as described by Mckone *et al.* [34]. ^¶^: changes in FEV_1_ % and BMI are the changes from previous visit (*i.e.* difference between measures 2 years prior to cystic fibrosis-related diabetes diagnosis and 1 year prior to CFRD diagnosis).

**FIGURE 2:**
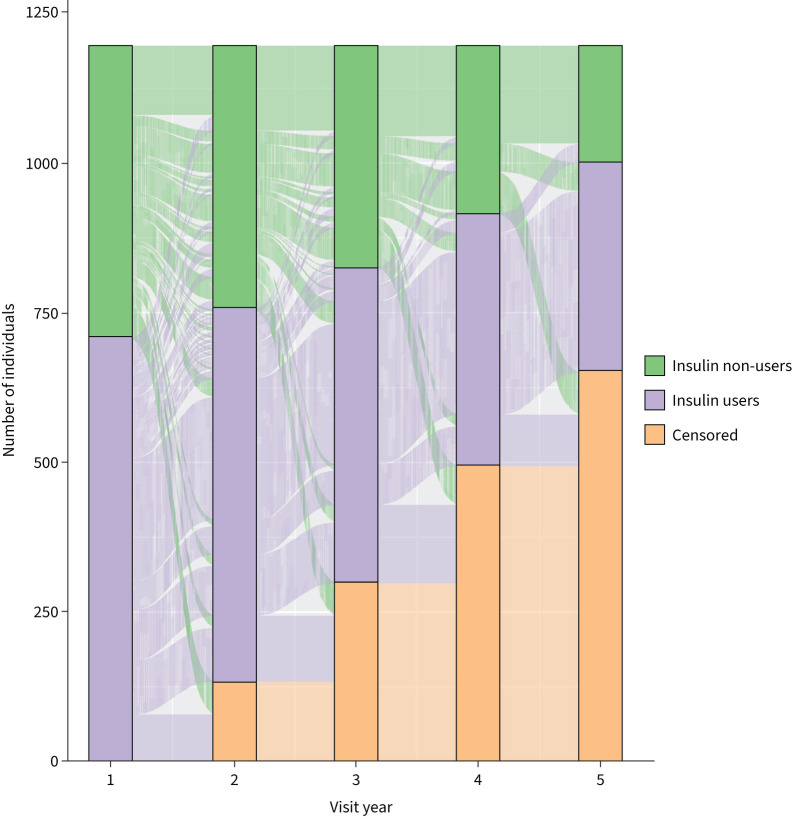
Flow of participants in each treatment group by follow-up year, for the n=1196 individuals included in the % predicted forced expiratory volume in 1 s (FEV_1_ %) analysis.

Figure 2 describes the flow of participants between treatment groups by year. Of the 222 individuals who were insulin users at baseline and had 5 years of follow-up, 206 (92.8%) remained on insulin for 5 years. Of the 324 individuals who did not use insulin at baseline and had 5 years of follow-up, 116 (35.8%) remained non-users for all 5 years.

### Lung function (FEV_1_ %) outcome

**FIGURE 3:**
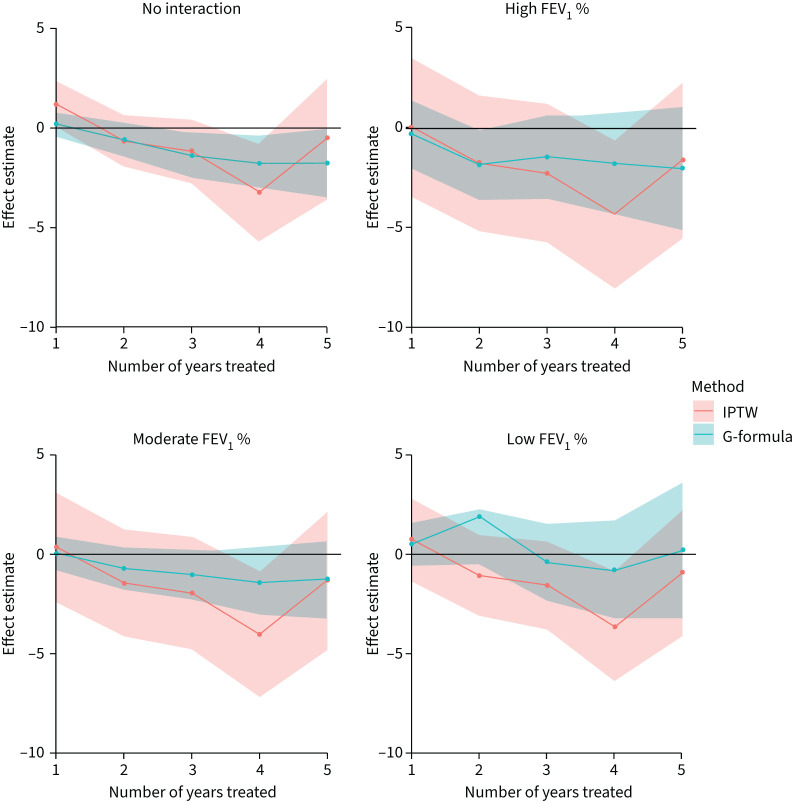
Estimated effects (and 95% confidence intervals) of insulin use for 1–5 years on % predicted forced expiratory volume in 1 s (FEV_1_ %) for the whole cohort (“no interaction”) and for people with high, moderate or low FEV_1_ % at baseline. High, moderate and low FEV_1_ % were defined as 100, 75 and 40, respectively. IPTW: inverse-probability-of-treatment weighting.

Figure 3 shows the estimated effects (and 95% CI) of insulin use for 1–5 years on FEV_1_ % from the two analysis methods. Corresponding numerical results are shown in supplementary table S3. Results are shown from a model that does not include an interaction between treatment and FEV_1_ % at baseline, therefore giving population average effect, and from a model including the interaction. For the model that incorporates the interaction we show results for people with high (FEV_1_ 100%), moderate (FEV_1_ 75%) and low (FEV_1_ 40%) lung function at baseline.

In the first year of insulin treatment there was evidence of a positive treatment effect on lung function (IPTW mean: FEV_1_ +1.3% (95% CI 0.05–2.44)) when compared to those not receiving insulin. Treatment effect estimates after the first year were negative (indicating worse lung function under insulin use), though CIs include 0 (except in year 4). There was no evidence that the treatment effect differed by FEV_1_ % at baseline (p=0.126), which is reflected in the small differences in treatment effects estimates between baseline FEV_1_ % levels in figure 3.

Results from the two analysis methods were very similar in terms of the point estimates; however the G-formula approach gave substantially narrower 95% CIs.

### BMI z-score outcome

**FIGURE 4:**
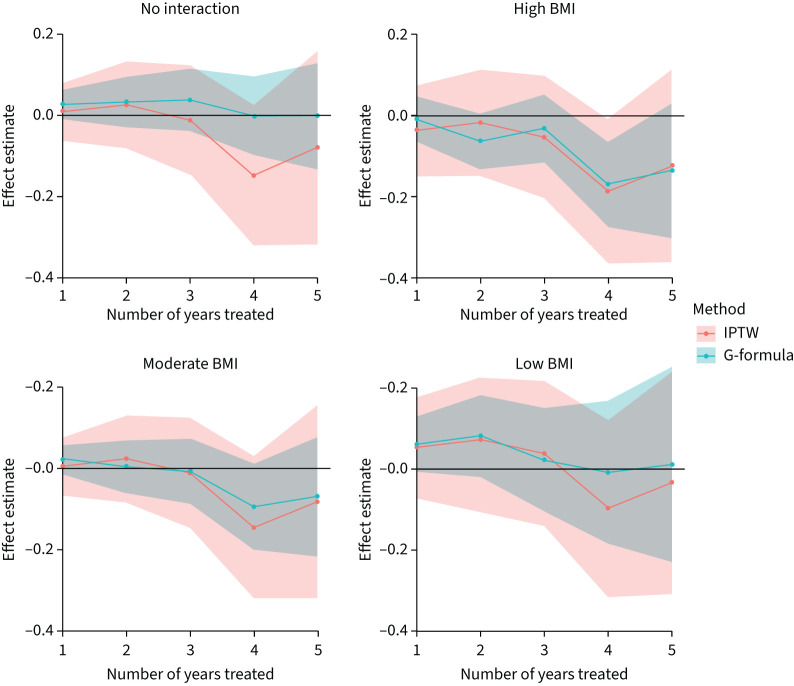
Estimated effects (and 95% confidence intervals) of insulin use for 1–5 years on body mass index (BMI) z-score for the whole cohort (“no interaction”) and for people with high, moderate or low BMI z-score at baseline. High, moderate and low BMI z-score was defined as the 80th, 50th and 20th percentiles of the distribution of BMI z-scores at baseline. IPTW: inverse-probability-of-treatment weighting.

Figure 4 shows the estimated effects (and 95% CI) of insulin use for 1–5 years on BMI z-score. Corresponding numerical results are shown in supplementary table S8. There is no evidence of an effect of insulin use on BMI z-score over 1–5 years since 0 is contained in the 95% CIs at each year.

Interestingly, estimated treatment effects were positive (in years 1–3) for people with lower baseline BMI, compared to being ∼0 for those with medium baseline BMI and negative for those with high baseline BMI. However, overall there was no evidence of an interaction between insulin use and baseline BMI (p=0.279).

Findings were similar from the two analysis methods, albeit with the G-formula approach giving more precise estimates.

### Sensitivity analyses

We conducted a number of sensitivity analyses to assess the impact of changes in our assumptions on our results. Details on the sensitivity analyses are provided in supplementary material section 4. Overall, the results were robust to a variety of different assumptions, including changes to how treatment history affects the outcome in the MSM and changes to directions of causal pathways.

## Discussion

In this study, we used national registry data to estimate the long-term treatment effects of insulin on clinical outcomes in incident CFRD. We found some evidence of short-term benefit to lung function and for BMI in certain scenarios, although overall there was little evidence to support a consistent longer-term benefit of insulin on lung function or BMI.

Insulin is currently the only recommended treatment in CFRD, given insulin deficiency is considered the primary aetiological process [1, 7, 8]. However, despite multiple national and international guidelines recommending its use, the evidence underpinning its effect on CF clinical outcomes remains weak. Only a handful of randomised controlled trials have been performed, with the two largest including 75 and 100 participants respectively [12, 13]. Overall, a recent Cochrane review and meta-analysis found no significant improvement in terms of lung function or BMI at 1 year [23].

The dearth of strong evidence in this area is likely, at least in part, related to the challenges of conducting prospective clinical trials in a condition which is itself a complication of a relatively rare disease, thus making recruitment to achieve sufficiently powered studies difficult. A variable disease course and heterogeneity in modalities and thresholds for diagnosis are further challenges. Given the challenges in this disease setting, it is unlikely clinical trials will be performed to understand the long-term impact of insulin. National registry data coupled with appropriate statistical methods enable estimation of causal effects of treatments, under strong assumptions. Earlier studies have demonstrated the potential for such methods in the CF setting [24], and there is an increasingly extensive literature on the application of these methods across different areas of clinical research.

The results from our study are consistent with the findings of the most recent Cochrane review, in that no significant treatment effect of insulin was seen on FEV_1_ or BMI after the first 24 months of treatment [14]. Our results also provide no evidence of a benefit of insulin on longer-term outcomes up to 5 years post-CFRD diagnosis. We found some suggestion that insulin could be more beneficial for individuals with low BMI. It could be postulated that those with lowest BMI are most insulin deficient and perhaps more likely to respond to insulin, thus suggesting insulin use in CFRD is more nuanced than guidelines currently suggest. In keeping with this, in the clinical trial by Ballmann *et al.* (where mean BMI z-score was much lower than in this study), a positive treatment effect for insulin was seen at 12 months [12].

Our results contrast with a number of earlier observational studies, where positive outcomes were associated with insulin use in CFRD [10, 11, 25, 26]. However, these studies are limited in that they all represent single-centre observational studies with little or no adjustment for potential confounding and often lacking control groups. In our sensitivity analyses, we found that when we did not adjust for potential confounders, insulin users tended to have lower FEV_1_ % and BMI at most follow-up times, which was attenuated after confounder adjustment (supplementary figures S10 and S16).

This study has three main advantages over previous observational studies. Firstly, our analyses included a far greater sample size than any study of insulin use in CFRD to date. Secondly, CF registries are well validated in providing standardised data over many years, allowing us to assess outcomes up to 5 years post insulin initiation [17]. Once initiated, insulin is often a lifelong therapy, and it is therefore important to understand long-term outcomes. To our knowledge this is the longest study of insulin in CFRD outcomes to date. Finally, we were able to adjust for potential confounders using state-of-the-art statistical methodology not previously applied in the CFRD setting, and similar findings were obtained using two analysis methods.

However, there are also limitations to this study. These include that diagnosis of CFRD is not completely standardised, with both continuous glucose monitoring (CGM) and oral glucose tolerance test (OGTT) recommended in the UK [7]. CGM is known to detect earlier dysglycaemia than OGTT, and heterogeneous use of these technologies between centres could create variation in incident diagnosis and subsequent treatment initiation [27]. The UK CF Registry does not record specific OGTT or CGM outcomes and hence evaluating the impact of such variation is challenging. Similar studies in established national CF registries where CGM use is less routine would be of great interest. Similarly, although the use of insulin is clearly recorded in the UK CF Registry, little is known about insulin regimens, adherence or how aggressively doses are uptitrated. There may be specific insulin regimes which do confer favourable clinical outcomes, yet we are unable to assess regimen-related difference in this study. We considered using HbA1c values as a surrogate measure of the effectiveness of insulin regimes; however HbA1c is not validated in this setting, and given there was substantial missingness on HbA1c data in the UK CF Registry (five-fold higher than our primary outcome data), we did not pursue this approach. When FEV_1_ % was the outcome of interest, we used FEV_1_ % measured on the day of the annual review. This ensured that the FEV_1_ % measurement used came after the initiation of insulin in insulin users. Finally, it is possible that there are additional confounders of the association between insulin use and the outcome that we did not account for and which could have resulted in biased treatment effect estimates.

The lack of evidence for a treatment effect on the outcomes considered in this study does not rule out other positive clinical effects for insulin in people with CFRD. Hyperglycaemia is linked to a wide range of deleterious diabetic and non-diabetic sequelae, and reducing hyperglycaemia has well-established positive outcomes in that regard. For example, even if insulin had no long-term effect on lung function or BMI, people with CFRD may still possibly derive benefit from insulin in terms of reduced bacterial chest infection and colonisation and vascular complications [3, 26, 28]. Similarly, microvascular complications of CFRD are prevalent in those with disease durations of >10 years, and our study period of 5 years precludes assessment of any longer-term benefits in that regard [29, 30]. A further longer-term consideration is the recent expansion in available CFTR modulators. CFTR modulators have been reported to improve glycaemia; however the extended disease duration and altered metabolic profile of people receiving long-term modulator therapy may have implications for diabetic complications and further work is needed in this area [31–33].

Equally, this study was unable to address possible transient short-term beneficial effects of insulin as the UK CF Registry data are collected (primarily) annually rather than on an encounter basis. Short-term improvements in lung function and BMI have been reported in a number of observational studies of insulin in CFRD. For example, a UK study has previously noted improved FEV_1_ and BMI at 3 months after insulin initiation but a return to baseline by 1 year [10]. In the context of the progressive lung function decline associated with CF, transient short-term improvements may still be important for people with CFRD if they act as a temporary pause on disease progression.

In conclusion, in this study we found no overall evidence of long-term improved clinical outcomes associated with insulin use in incident CFRD. Given the lack of long-term benefits seen here, and high treatment burden associated with insulin, future work should explore the use of alternative hypoglycaemic agents in CFRD, while precisely defining who is likely to benefit from insulin treatment.

## Supplementary material

10.1183/23120541.00170-2022.Supp1 **Please note:** supplementary material is not edited by the Editorial Office, and is uploaded as it has been supplied by the author.
Supplementary material 00170-2022.SUPPLEMENT
