# Supplementary material for: The long-term effects of insulin use in incident cystic fibrosis-related diabetes: a target trial emulated using longitudinal national registry data
Source: ERJ Open Res. 2022 Nov 7;8(4):00170-2022. doi: 10.1183/23120541.00170-2022 (PMC9638829; doi:10.1183/23120541.00170-2022)

2SUPPLEMENTARY MATERIAL

THE LONG-TERM EFFECTS OF INSULIN USE IN INCIDENT CYSTIC FIBROSIS RELATED DIABETES. A TARGET-TRIAL EMULATED USING LONGITUDINAL NATIONAL REGISTRY DATA

Emily Granger[1], Ruth H. Keogh[1], Freddy Frost[2]

[1]*Department of Medical Statistics,*
Faculty of Epidemiology and Population Health,
London School of Hygiene and Tropical Medicine,
Keppel Street, London, WC1E7HT

[2]*Institute of Infection, Veterinary and Ecological Sciences,*
University of Liverpool,
Liverpool, UK

This document consists of four sections: (1) Additional notes on methodology, (2) Details about missing data, (3) Details on the number of individuals censored by year, and (4) Additional results from sensitivity analyses that supplement the results given in the main manuscript. The current document is organised as follows:

1. *Additional notes on methodology*
    - *1.1 Causal effects of interest*
    - *1.2 Confounding variables*
    - *1.3 Inverse-probability-of-treatment weighting of marginal structural models.*
    - *1.4 G-formula*
    - *1.5 Sensitivity analyses*
    - *1.6 Assessing the impact of confounding bias*
2. *Missing data*
    - *2.1 Amount of missing data*
    - *2.2 Missingness patterns*
    - *2.3 Methods for handling missing data*
3. *Censoring*
4. *Additional results*
    - *4.1 Outcome trajectories*
    - *4.2 FEV1% as outcome*
        - *4.2.1 Distribution of weights*
        - *4.2.2 Tabulated values for the models with an interaction term*
        - *4.2.3 Sensitivity to model specification*
        - *4.2.4 Sensitivity to causal pathways*
        - *4.2.5 Including rate of decline of lung function as a time-varying confounder*
        - *4.2.6 Unadjusted analyses*
    - *4.3 BMI z-score as outcome*
        - *4.3.1 Distribution of weights*
        - *4.3.2 Tabulated values for the models with an interaction term*
        - *4.3.3 Sensitivity to model specification*
        - *4.3.4 Sensitivity to causal pathways*
        - *4.3.5 Including rate of decline of lung function as a time-varying confounder*
        - *4.3.6 Unadjusted analyses*

1. Additional notes on methodology

1.1 Causal effects of interest

Time is measured in years since CFRD diagnosis. We let A_k denote treatment (insulin) use at time k, with $A_k = 1$ denoting insulin use and $A_k = 0$ denoting not using insulin. The outcome at time k is denoted Y_k. Let $\overline{A}_k = (a_1, \dots, a_k)$ denote the treatment history from time point 1 (i.e. after 1 year of treatment) to time point k (after k years of treatment) and let $Y_k^{\overline{A}_k}$ denote the potential outcome that would be observed for an individual with treatment history $\overline{A}_k$. The treatment effects of interest are then defined as:

1 year:	$E\left(Y_1^{\bar{A}_1=1}\right) - E\left(Y_1^{\bar{A}_1=0}\right)$
2 year:	$E\left(Y_2^{\bar{A}_2=(1,1)}\right) - E\left(Y_2^{\bar{A}_2=(0,0)}\right)$
3 year:	$E\left(Y_3^{\bar{A}_3=(1,1,1)}\right) - E\left(Y_3^{\bar{A}_3=(0,0,0)}\right)$
4 year:	$E\left(Y_4^{\bar{A}_4=(1,1,1,1)}\right) - E\left(Y_4^{\bar{A}_4=(0,0,0,0)}\right)$
5 year:	$E\left(Y_5^{\bar{A}_5=(1,1,1,1,1)}\right) - E\left(Y_5^{\bar{A}_5=(0,0,0,0,0)}\right)$

We were also interested in the conditional treatment effects, conditional on individuals having either high, moderate or low outcomes (FEV1% and BMI z-score) at baseline, where baseline is time 0 (the year prior to CFRD diagnosis where no individuals are on treatment). For each year, we estimated the following treatment effects:

$$E\left(Y_k^{\bar{A}_k=\bar{1}}|Y_0 = High\right) - E\left(Y_k^{\bar{A}_k=\bar{0}}Y_0 = High\right)$$

$$E\left(Y_k^{\bar{A}_k=\bar{1}}|Y_0 = Moderate\right) - E\left(Y_k^{\bar{A}_k=\bar{0}}|Y_0 = Moderate\right)$$

$$E\left(Y_k^{\bar{A}_k=\bar{1}}|Y_0 = Low\right) - E\left(Y_k^{\bar{A}_k=\bar{0}}|Y_0 = Low\right)$$

Where Y_0 is the outcome at baseline. High, moderate and low FEV_1% was defined as 100, 75 and 40, respectively. High, moderate and low BMI z-score was defined as the 80th, 50th and 20th percentiles of the distribution of BMI z-scores at baseline, these were 0.83, -0.10 and -1.18 respectively.

1.2 Confounding variables

To obtain unbiased estimates of the treatment effects, we needed control for both time-invariant confounders (sex, age, genotype, FEV_1% at baseline) and time-varying confounding (*Pseudomonas aeruginosa*, *Burkholderia cenocepacia complex*, and pancreatic insufficiency [measured by use of pancreatic enzyme supplements]). Genotype was classed as either high risk, low risk or not assigned as previously[1]. The assumptions we made regarding the relationships between exposure, outcome and confounders in the analyses with FEV_1% as outcome are given in the directed acyclic graph (DAG) Figure S.1

Figure S. 1: Directed Acyclic Graph depicting the assumed relationships between variables in the analysis

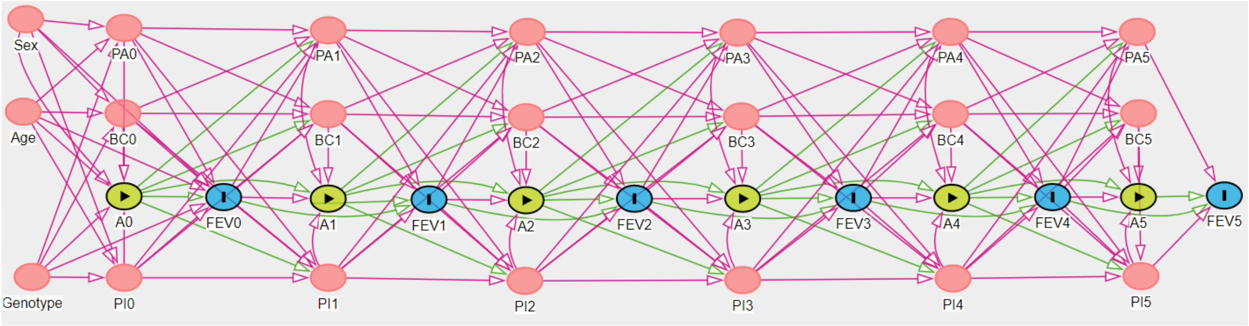

PAk: Pseudomonas aeruginosa at time point k, BCk: Burkholderia cenocepacia at time point k; Ak: Treatment at time point k; FEVk: FEV_1% at time point k; PIk; pancreatic insufficiency at time point k. We assume that age, sex and genotype also affect all variables from time 1 onwards, but these arrows are omitted for simplicity.

In the BMI z-score analyses, two additional confounders were included: BMI at baseline (time-invariant) and nutritional support (time-varying). Given the population of this study included children (where age-adjusted z-scores are routinely reported), and adults (where raw BMI is reported), we utilised the age-20 BMI z-score reference for all individuals 20 years and older to generate a standardised metric across age ranges. Nutritional support was defined as supplementary feeding by jejunal tube, nasogastric tube, gastrostomy or parenteral administration, as have been previously defined.[1]

In both analyses we also adjusted for the rate of change in FEV_1% observed prior to CFRD diagnosis. We defined the following linear mixed model with random slope and intercept:

$$FEV_1\%_{ij} = (\alpha_0 + \delta_{0i}) + (\alpha_1 + \delta_{1i})j + e_{ij}$$

Where $j \in \{0,1,2,3,4\}$ is the number of years before baseline ($j = 0$ is the baseline year) and $FEV_1\%_{ij}$ is the percent predicted forced expiratory volume for the ith individual at time j. The estimate of the slope parameter $(\alpha_1 + \delta_{1i})$ for each individual is used as a time-invariant variable representing rate of change in FEV_1%.

1.3 Inverse-probability-of-treatment weighting of marginal structural models.

Let $\boldsymbol{L_B}$ denote the set of time-invariant confounders and $\boldsymbol{L_k}$ denote the set of time-varying confounders at time k (given in Section 1.2). Additional to the time-varying variables stated in Section 1.2, $\boldsymbol{L_k}$ includes the lagged outcome, where lagged outcome at time *k* equals the outcome at time *k-1*.

Then, the stabilised inverse-probability-of-treatment weights for individual i at time K were defined as:

$$IPT.w_{iK} = \frac{\prod_{k=0}^{K} \Pr(A_k = a_{ik} | \bar{A}_{k-1} = a_{ik-1}, \boldsymbol{L_B} = \boldsymbol{l_i})}{\prod_{k=0}^{K} \Pr(A_k = a_{ik} | \bar{A}_{k-1} = a_{ik-1}, \boldsymbol{L_B} = \boldsymbol{l_i}, \boldsymbol{\bar{L}_k} = \boldsymbol{\bar{l}_{ik}})}$$

Weights were also used to account for missing outcome data ($MISS.w_{iK}$), loss-to-follow-up ($LTFU.w_{iK}$) censoring due to death or transplant ($CENS.w_{iK}$) and time-varying eligibility due to use of CFTR modulators or oral corticosteroids ($ELIG.w_{iK}$).

[1] McKone E, Goss C, Aitken M. CFTR Genotype as a Predictor of Prognosis in Cystic Fibrosis. *Chest* 2006; 130: 1141–1147.

The probabilities required for each set of weights were obtained using logistic regression. For the $IPT.w_{iK}$ and $ELIG.w_{iK}$, the outcomes were indicators of treatment and eligibility, respectively.

For each individual, we included all time points with observed outcome data in the analysis. To account for the missing outcome data, the included data were re-weighted by the inverse of their probability of remaining in the study, conditional on treatment and confounder history. The weights, $MISS.w_{iK}$ were estimated using a similar equation as the one for $IPT.w_{iK}$, but the outcome was an indicator for missingness in the outcome for the ith individual at time *K*.

Individuals who were lost to follow-up, died or had an organ transplant were censored (i.e. they could not re-enter the study). For the censoring weights, the outcome at time *k* was an indicator for whether the individual was censored at time *k+1*.

The final weight for individual i at time point K ($FINAL.w_{iK}$) was defined as a product of all of the above weights:

$$FINAL.w_{iK} = IPT.w_{iK} \times MISS.w_{iK} \times LTFU.w_{iK} \times CENS.w_{iK} \times ELIG.w_{iK}$$

We then fitted the following marginal structural models (MSM) in the weighted sample

Model 1:
(without interaction)

$$Y_{ik}^{\bar{A}_k} = \beta_0 + \beta_1 A_{ik} + \sum_{c=2}^{5} \boldsymbol{\beta_c I(k=c)} + \boldsymbol{\beta_3 L_{Bi}} + \sum_{c=2}^{5} \boldsymbol{\beta_{4c} I(k=c)}\, \boldsymbol{A_{ik}} + \varepsilon_{ik}$$

Model 1b:
(with interaction)

$$Y_{ik}^{\bar{A}_k} = \beta_0 + \beta_1 A_{ik} + \sum_{c=2}^{5} \boldsymbol{\beta_c I(k=c)} + \boldsymbol{\beta_3 L_{Bi}} + \sum_{c=2}^{5} \boldsymbol{\beta_{4c} I(k=c)}\, \boldsymbol{A_{ik}} + \beta_5 A_{ik} \times Y_0 + \varepsilon_{ik}$$

Time, sex and genotype were modelled as factors. Age was modelled using regression splines with knots at the 25th, 50th and 75th percentiles of the distribution of baseline age. Models 1 and 1b assume that the outcome at time k depends only on treatment at time k. We have considered additional MSMs which relax this assumption (defined in Section 1.5).

Model 1 was fitted using IPTW to obtain estimates of the unconditional causal effects and Model 1b was used to obtain estimates of the conditional causal effect estimates (defined in Section 1.1).

1.4 G-formula

The G-formula is an alternative approach to fitting the same marginal structural models that are specified above (Section 1.3). Using the G-formula requires specification of models for the time-varying confounders as well as treatment. Let $\boldsymbol{Z_k} = (A_k, C_k, \boldsymbol{L_k})$, where A_k denotes treatment use at time *k*, C_k denotes whether an individual was censored at time k (due to loss-to-follow up, transplant, death, use of oral corticosteroids or CFTR modulators or missing outcome data) and $\boldsymbol{L_k}$ denotes the set of time-varying confounders at time *k*. Then, the G-formula is given by:

$$E\left(Y_k^{\bar{A}_k}\right) = \sum_{\bar{\boldsymbol{l}}} E[Y_k | \bar{A}_k = \bar{a}_k, \bar{C}_k = \bar{0}, \bar{\boldsymbol{L}}_k = \bar{\boldsymbol{l}}, \boldsymbol{L_B} = \boldsymbol{l}] \prod_{j=0}^{k} P(\boldsymbol{z}_j | \bar{a}_{j-1}, c_{j-1} = 0, \bar{\boldsymbol{l}}_{j-1,} \boldsymbol{l})$$

Where $P(\boldsymbol{z}_k | \bar{a}_{k-1}, c_{k-1} = 0, \bar{\boldsymbol{l}}_{k-1,} \boldsymbol{l})$ is the conditional joint distribution of $\boldsymbol{Z_k}$. The G-formula can be used to estimate the expected potential outcome when $\bar{A} = \bar{a}$.

Let PI_k, BC_k, PA_k and lag_FEV_k denote pancreatic insufficiency, *Burkholderia cenocepacia*, *Pseudomonas aeruginosa* at time *k*, and FEV_1% at time *k-1,* respectively (these are the variables in

$\boldsymbol{L_k}$). These time-varying confounders were modelled using logistic regression for the binary variables (PI_k, BC_k, PA_k) and linear regression for the continuous variable (lag_FEV_k) to obtain the conditional probabilities needed for the g-formula:

$$logit\big(P(PI_k = 1)\big) = \gamma_0 + \gamma_1 PI_{k-1} + \gamma_2 BC_{k-1} + \gamma_3 PA_{k-1} + \gamma_4 lag\_FEV_k + \gamma_5 A_{k-1} + \boldsymbol{\gamma_6 L_B} + \varepsilon_k$$

$$logit\big(P(BC_k = 1)\big) = \gamma_0 + \gamma_1 PI_{k-1} + \gamma_2 BC_{k-1} + \gamma_3 PA_{k-1} + \gamma_4 lag\_FEV_k + \gamma_5 A_{k-1} + \boldsymbol{\gamma_6 L_B} + \varepsilon_k$$

$$logit\big(P(PA_k = 1)\big) = \gamma_0 + \gamma_1 PI_{k-1} + \gamma_2 BC_{k-1} + \gamma_3 PA_{k-1} + \gamma_4 lag\_FEV_k + \gamma_5 A_{k-1} + \boldsymbol{\gamma_6 L_B} + \varepsilon_k$$

$$lag\_FEV_k = \gamma_0 + \gamma_1 PI_{k-1} + \gamma_2 BC_{k-1} + \gamma_3 PA_{k-1} + \gamma_4 lag\_FEV_{k-1} + \gamma_5 A_{k-1} + \boldsymbol{\gamma_6 L_B} + \varepsilon_k$$

$$logit\big(P(A_k = 1)\big) = \gamma_0 + \gamma_1 PI_k + \gamma_2 BC_k + \gamma_3 PA_k + \gamma_4 lag\_FEV_k + \gamma_5 A_{k-1} + \boldsymbol{\gamma_6 L_B} + \varepsilon_k$$

The censoring indicator (C_k) was modelled using logistic regression:

$$logit\big(P(C_k = 0 | C_{k-1} = \bar{0})\big) = \gamma_0 + \gamma_1 PI_{k-1} + \gamma_2 BC_{k-1} + \gamma_3 PA_{k-1} + \gamma_4 lag\_FEV_k + \gamma_5 A_{k-1} + \boldsymbol{\gamma_6 L_B} + \varepsilon_k$$

Outcomes were modelled using linear regression:

Model 1: (without interaction)

$$Y_{ik} = \beta_0 + \beta_1 A_{ik} + \sum_{c=2}^{5} \boldsymbol{\beta_c I(k = c)} + \boldsymbol{\beta_3 L_{Bi}} + \sum_{c=2}^{5} \boldsymbol{\beta_{4c} I(k = c)}\, \boldsymbol{A_{ik}} + \boldsymbol{\beta_5} \bar{L}_k + \varepsilon_{ik}$$

Model 1b: (with interaction)

$$Y_{ik} = \beta_0 + \beta_1 A_{ik} + \sum_{c=2}^{5} \boldsymbol{\beta_c I(k = c)} + \boldsymbol{\beta_3 L_{Bi}} + \sum_{c=2}^{5} \boldsymbol{\beta_{4c} I(k = c)}\, \boldsymbol{A_{ik}} + \boldsymbol{\beta_5} \bar{L}_k + \beta_6 A_{ik} \times Y_0 + \varepsilon_{ik}$$

The above models were those used in the analysis with $FEV_1\%$ as the outcome. When BMI z-score was the outcome, nutritional support and BMI z-score were additionally included as time-varying confounders.

1.5 Sensitivity analyses

We conducted sensitivity analyses to assess the impact on our results of (1) changing the specification of the marginal structural models, (2) changing the direction of causal pathways in the DAG, and (3) including rate of decline in $FEV_1\%$ as a time-varying confounder in the analyses.

(1) Marginal structural models

Both the IPTW and G-formula analyses were repeated using two additional marginal structural model for potential outcomes:

Model 2:

$$Y_{ik}^{\bar{A}_{ik}} = \beta_0 + \boldsymbol{\beta_1} \sum_{j=0}^{k} A_{ij} + \sum_{c=2}^{5} \boldsymbol{\beta_c I(k = c)} + \boldsymbol{\beta_3 L_{Bi}} + \varepsilon_{ik}$$

Model 3:

$$Y_{ik}^{\bar{A}_{ik}} = \beta_0 + \sum_{j=0}^{k} \beta_j A_{ij} + \sum_{c=2}^{5} \boldsymbol{\beta_c I(k = c)} + \boldsymbol{\beta_3 L_{Bi}} + \varepsilon_{ik}$$

where $\sum_{j=0}^{k} A_{ij}$ is the cumulative treatment observed for individual i at time point k.

These models were fitted using IPTW and g-formula to estimate treatment effects for years 1-5 (defined in Section 1.1)

(2) Changes to the DAG

The DAG in Figure S.1 makes assumptions about the direction of causal pathways. For some variables, the correct direction of causal pathway is clear. For example, the causal pathway between treatment use over the past year and BMI or lung function measured on the day is clear due to the temporal ordering. However, for some of our variables, the appropriate direction of causal pathway is less clear. For example, our treatment variable and time-varying confounder variables are summaries of the previous year and it is not always clear if treatment, or confounding variable, was observed first. We have made the assumption that time-varying confounders at time *k* influence treatment use at time *k*. To assess the sensitivity of our results to changes in the direction of causal pathways, we repeated the g-formula analyses, making a different assumption that treatment use at time *k* influences time-varying confounders at time *k*. This changes the conditional models used in the g-formula analysis to:

$$logit\big(P(PI_k = 1)\big) = \gamma_0 + \gamma_1 PI_{k-1} + \gamma_2 BC_{k-1} + \gamma_3 PA_{k-1} + \gamma_4 lag\_FEV_k + \gamma_5 A_k + \boldsymbol{\gamma_6 L_B} + \varepsilon_k$$

$$logit\big(P(BC_k = 1)\big) = \gamma_0 + \gamma_1 PI_{k-1} + \gamma_2 BC_{k-1} + \gamma_3 PA_{k-1} + \gamma_4 lag\_FEV_k + \gamma_5 A_k + \boldsymbol{\gamma_6 L_B} + \varepsilon_k$$

$$logit\big(P(PA_k = 1)\big) = \gamma_0 + \gamma_1 PI_{k-1} + \gamma_2 BC_{k-1} + \gamma_3 PA_{k-1} + \gamma_4 lag\_FEV_k + \gamma_5 A_k + \boldsymbol{\gamma_6 L_B} + \varepsilon_k$$

$$lag\_FEV_k = \gamma_0 + \gamma_1 PI_{k-1} + \gamma_2 BC_{k-1} + \gamma_3 PA_{k-1} + \gamma_4 lag\_FEV_{k-1} + \gamma_5 A_{k-1} + \boldsymbol{\gamma_6 L_B} + \varepsilon_k$$

$$logit\big(P(A_k = 1)\big) = \gamma_0 + \gamma_1 PI_{k-1} + \gamma_2 BC_{k-1} + \gamma_3 PA_{k-1} + \gamma_4 lag\_FEV_k + \gamma_5 A_{k-1} + \boldsymbol{\gamma_6 L_B} + \varepsilon_k$$

(3) Including rate of decline in FEV_1% as a time-varying confounder

In Section 1.2 we described how we obtained an estimate of the rate of change of FEV_1% prior to CFRD diagnosis, using the five years prior to diagnosis. This was then included in our analyses as a time-invariant confounder. When including it as a time-varying confounder, we repeated the same analysis on different subset of years. When moving forward by one time point, the subset of years used in the analysis to estimate the slope was shifted forward by one year. This resulted in a variable which gives the estimated rate of change of FEV_1% at each time point.

1.6 Assessing the impact of confounding bias

Finally, we assessed the impact of confounding bias (by measured confounders) in the results by conducting unadjusted and partially adjusted analyses.

The partially adjusted analyses adjusted for time-invariant confounders only. For IPTW, this was done by setting all weights to 1 and including the time-invariant confounders in the marginal structural model. When using the G-formula (defined in Section 1.4), the set of time-varying confounders was empty ($\boldsymbol{L_k} = \emptyset$**).**

The unadjusted analyses did not adjust for any confounders. For IPTW, this was done by setting all weights to 1 and not including any time-invariant confounders in the marginal structural model (i.e. the outcome was modelled using current treatment and time only). When using the G-formula, the set of time-varying and time-invariant confounders were empty ($\boldsymbol{L_k} = \boldsymbol{L_B} = \emptyset$**).**

2. Missing data

2.1 Amount of missing data

We found 1613 individuals aged 12 years and over who were diagnosed with CFRD between 2008 and 2016. After excluding transplant recipients, and people receiving CFTR modulators or oral corticosteroids at baseline, 1298 individuals remained. Table 1 shows the amount of missing data for those 1298 individuals in all variables in our analysis, by year. The number of individuals decreases by year due to death or lost to follow-up.

There was no missing data in our exposure variable (insulin use), sex, or baseline age. The variable with the most missing data was FEV_1%, where each year between 4.1-4.7% of FEV_1% data were missing.

Table 1: Amount of missing data by year

Year (k):	0 (n=1298)	1 (n=1298)	2 (n=1107)	3 (n=935)	4 (n=768)	5 (n=627)
Insulin use	0 (0.0%)	0 (0.0%)	0 (0.0%)	0 (0.0%)	0 (0.0%)	0 (0.0%)
Sex	0 (0.0%)	NA	NA	NA	NA	NA
Baseline Age	0 (0.0%)	NA	NA	NA	NA	NA
Genotype	11 (0.8%)	NA	NA	NA	NA	NA
FEV_1%	55 (4.2%)	58 (4.5%)	47 (4.2%)	38 (4.1%)	36 (4.7%)	29 (4.6%)
BMI z-score	26 (2.0%)	27 (2.1%)	24 (2.2%)	16 (1.7%)	18 (2.3%)	16 (2.6%)
P. aeruginosa infection	0 (0.0%)	3 (0.2%)	1 (0.1%)	2 (0.2%)	4 (0.5%)	4 (0.6%)
B.cenocepacia complex infection	0 (0.0%)	3 (0.2%)	1 (0.1%)	2 (0.2%)	4 (0.5%)	4 (0.6%)
Pancreatic enzyme use	28 (2.2%)	33 (2.5%)	24 (2.2%)	15 (1.6%)	14 (1.8%)	10 (1.6%)
Nutritional support	0 (0.0%)	1 (0.1%)	0 (0.0%)	0 (0.0%)	0 (0.0%)	1 (0.2%)

2.2 Missingness patterns

Figure S.3 shows pair plots which depict the relationships between missing values and observed values in all variables. For example, the plot in row 2, column 1, compares the distribution of FEV_1% at baseline among individuals with observed BMI (blue boxplot) and the distribution of FEV_1% at baseline among individuals with missing BMI (grey boxplot). These plots are useful for informing whether data is missing completely at random (MCAR) or missing at random (MAR). Overall, the trends do not indicate strong violations against MCAR as missingness does not appear to depend on the observed data.

Figure S. 2: Comparing the distribution of variables in the data between missing and observed values in all other variables.

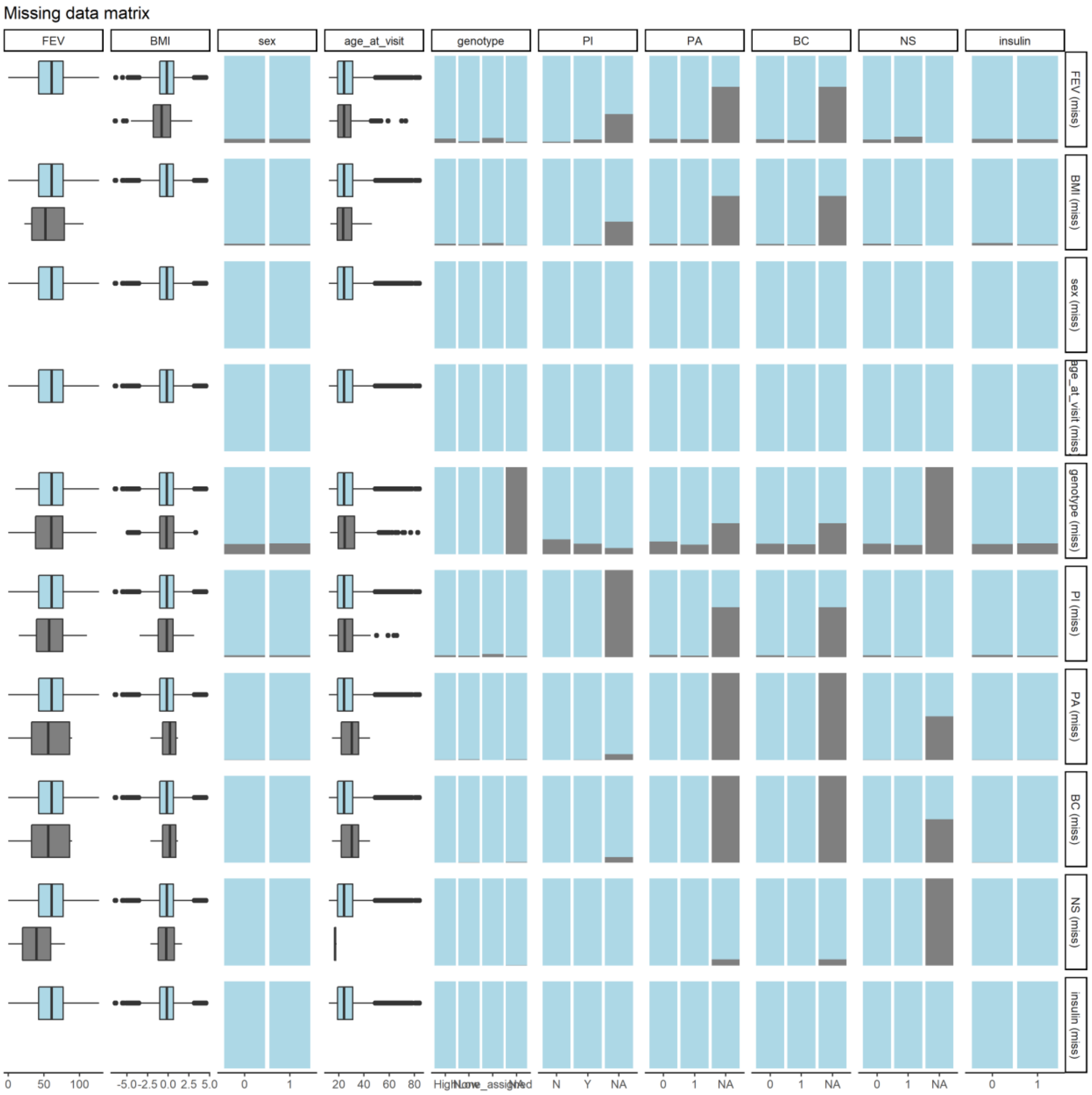

** The blue regions show the distribution of the column variable across individuals with observed data in the row variable. The grey regions show the distribution of the column variable across individuals with missing data in the row variable. Equal distributions indicate data are missing completely at random (i.e. do not depend on observed data). PI: Pancreatic Insufficiency; PA: Pseudomonas Aeruginosa; BC: Burkholderia cenocepacia; NS: Nutritional Support*

2.3. Methods for handling missing data

Initially, a simple imputation method was used to deal with missing data on pancreatic insufficiency. Pancreatic enzyme supplement use is usually static over time (i.e. used for long-term), and so for missing years where the prior year and subsequent year were equal (either recorded as use or non-use in both years), we assumed the missing value would be the same. Originally, there were 181 individuals with missing data on pancreatic enzyme supplement use; after implementing our imputation method, this reduced to 40.

After using the above approach to deal with some of the missing data on pancreatic insufficiency, individuals who still had missing data on any of the confounders (time-invariant or time-varying at any time point) where excluded from the analysis. A total of 50 individuals were excluded due to missing genotype, infection data or data on pancreatic insufficiency. 52 individuals were excluded due to missing data for baseline FEV_1%. In the analysis with BMI as the outcome, a further 4 individuals were excluded due to missing data for baseline BMI.

Missing outcome data were handled using weights as described in Section 1.3. The number of individuals with missing outcome data by follow-up year are given in Section 3, Tables S.2 and S.3.

3. Summary of exclusions due to lost to follow-up, death, transplant, ineligibility and missing outcome.

Tables S.2 and S.3 give the numbers of people who were excluded or censored each year due to different reasons in the FEV_1% and BMI analysis, respectively. People who were censored due to loss-to-follow-up, death or transplant, did not re-enter the study and these individuals account for the decreasing sample size by year. People who were temporarily excluded due to missing data or temporary ineligibility (due to initiating treatment with CFTR modulators or oral corticosteroids) were allowed to re-enter the study, and these numbers account for the differences between the number of people in each follow up year (row 1) and the number of people included in the final analysis (row 6). In the inclusion criteria, we required people to have at least one year of follow-up, hence there are no deaths, transplants or loss-to-follow-up (LTFU) in year 1.

Table S.2: Number of people censored for different reasons by year in the analysis with FEV_1% as the outcome

Follow-up year:	1 (N=1196)	2 (N=1062)	3 (N=898)	4 (N=702)	5 (N=546)
LTFU	0	109	133	174	146
Death or tx	0	25	31	22	10
Ineligible	0	88	113	93	80
Missing FEV_1%	77	39	58	44	32
Final N FEV_1%	1119	935	727	565	434

LTFU: Lost to follow-up; tx: transplant; ineligible: temporarily censored due lack of ineligibility (i.e. started treatment with CFTR modulators or oral corticosteroids); Missing FEV_1%: temporarily excluded due to missing FEV_1% data; Final N FEV_1%: final number of people included in the analysis after censoring

Table S.3: Number of people censored for different reasons by year in the analysis with BMI as the outcome

Follow-up year:	1 (N=1192)	2 (N=1058)	3 (N=895)	4 (N=699)	5 (N=544)
LTFU	0	109	132	174	145
Death or tx	0	25	31	22	10
Ineligible	0	88	113	93	80
Missing data	86	20	44	34	26
Final N BMI	1106	950	738	572	438

LTFU: Lost to follow-up; tx: transplant; ineligible: temporarily censored due lack of ineligibility (i.e. started treatment with CFTR modulators or oral corticosteroids); Missing data: temporarily excluded due to missing data (on BMI or FEV_1%); Final N BMI: final number of people included in the analysis after censoring

4. Additional results

4.1 Outcome trajectories

Figures S.4 shows the average outcomes (FEV_1% and BMI z-score) in the analysis cohort, by follow-up year. The average FEV_1% decreases by year, whereas the average BMI z-score tends to increase by year.

Figure S. 3: Average FEV_1% and BMI z-score in the whole cohort, by follow-up year

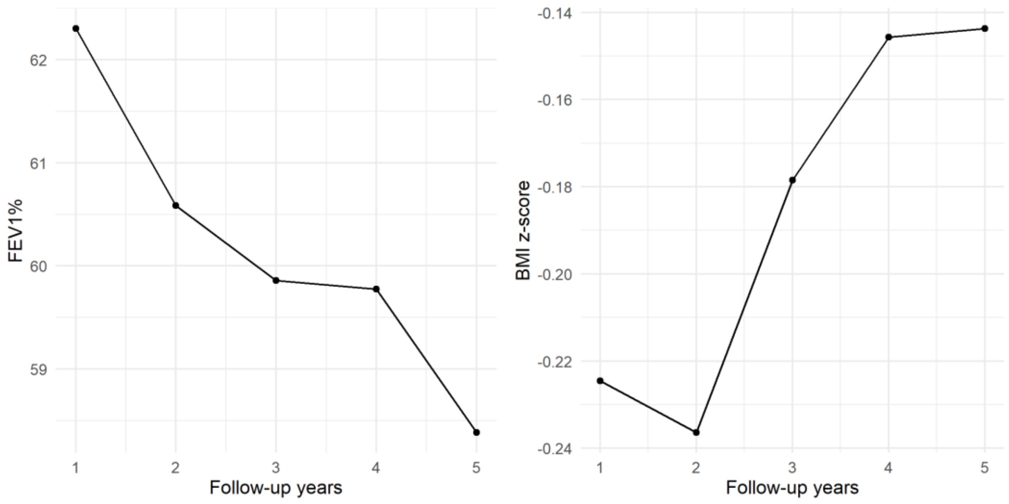

4.2 FEV_1% as outcome

4.2.1 Distribution of weights

Figures S.5 and S.6 show the distribution of inverse-probability-of-treatment weights and overall weights by year, respectively. Boxplots show that the median weights are approximately 1 for each year, as expected. The variance of weights increases by year but there are no extreme values.

Figure S. 4: Distribution of inverse-probability-of-treatment weights by year. Horizontal line at y=1.

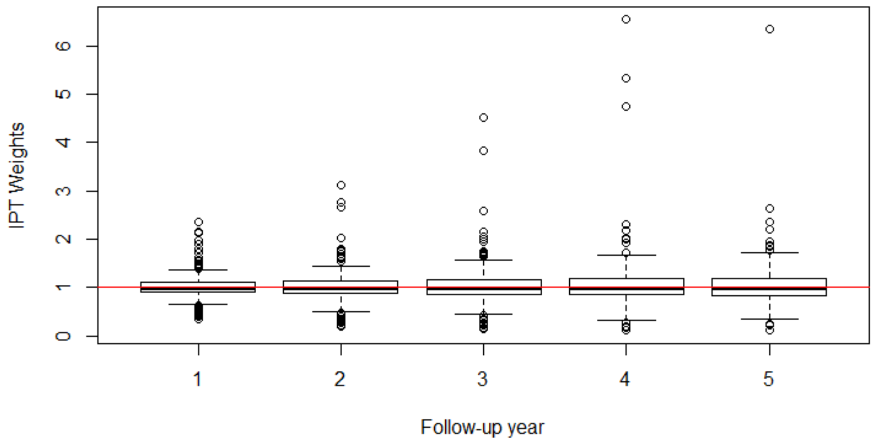

Figure S. 5: Distribution of overall weights by year. Horizontal line at y=1.

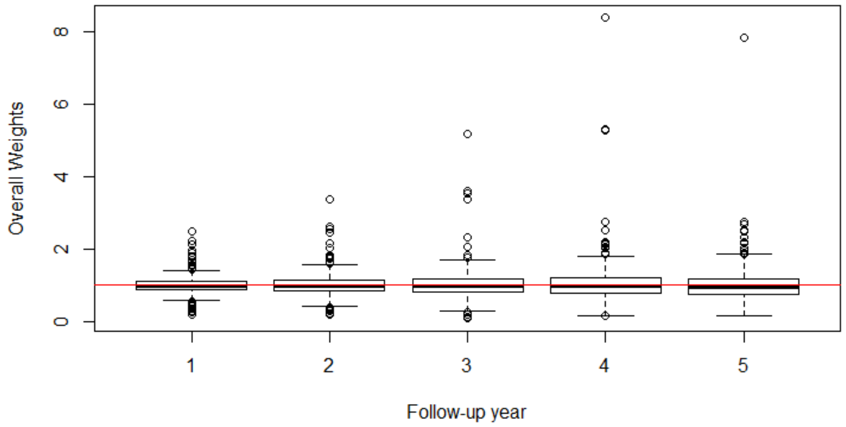

4.2.2 Tabulated results corresponding to main text Figure 3

Table S.4 shows the estimated effects of insulin use for 1-5 years on FEV_1% for the whole cohort, and conditional on high, moderate or low FEV_1% at baseline. The estimated effects for the whole cohort are obtained using Model 1 and the conditional effects are estimated using Model 1b, as defined in Section 1.3. For both the IPTW and G-formula results, estimated effects tend to increase when baseline FEV_1% decreases.

Table S.4: Estimates of the effect of insulin use for 1-5 years on FEV_1% for the whole cohort, and conditional on high (100), moderate (75) or low (45) FEV_1% at baseline, obtained using inverse-probability-of-weighting (IPTW) and the G-Formula

Year	FEV_1%	IPTW	G-formula
1	Whole cohort	1.25 (0.05, 2.44)	0.22 (-0.38, 0.82)
	100	0.39 (-3.09, 3.87)	-0.27 (-1.98, 1.43)
	75	0.60 (-2.15, 3.36)	0.05 (-0.77, 0.87)
	40	0.90 (-1.18, 2.98)	0.50 (-0.56, 1.57)
2	Whole cohort	-0.50 (-1.79, 0.79)	-0.59 (-1.43, 0.26)
	100	-1.35 (-4.75, 2.06)	-1.82 (-3.59, -0.05)
	75	-1.13 (-3.83, 1.57)	-0.72 (-1.78, 0.34)
	40	-0.84 (-2.88, 1.21)	0.88 (-0.51, 2.28)
3	Whole cohort	-1.11 (-2.75, 0.53)	-1.37 (-2.53, -0.21)
	100	-1.95 (-5.44, 1.54)	-1.43 (-3.52, 0.65)
	75	-1.74 (-4.56, 1.09)	-1.04 (-2.28, 0.20)
	40	-1.44 (-3.67, 0.80)	-0.41 (-2.37, 1.55)
4	Whole cohort	-3.23 (-5.74, -0.71)	-1.77 (-3.18, -0.36)
	100	-4.05 (-7.76, -0.34)	-1.75 (-4.30, 0.80)
	75	-3.84 (-7.02, -0.66)	-1.43 (-3.05, 0.18)
	40	-3.54 (-6.31, -0.78)	-0.77 (-3.23, 1.70)
5	Whole cohort	-0.55 (-3.64, 2.55)	-1.75 (-3.48, -0.02)
	100	-1.38 (-6.31, -0.78)	-2.00 (-5.11, 1.11)
	75	-1.17 (-4.66, 2.33)	-1.27 (-3.21, 0.68)
	40	-0.87 (-4.05, 2.31)	0.20 (-3.23, 3.63)

4.2.3 Sensitivity to marginal structural model specification

Table S.5 and Figure S.7 show the estimated effects of insulin use for 1-5 years on FEV_1% obtained using Models 1-3 (defined in Section 1.5). For the G-formula, results were very similar between model specifications. There were some differences in the trends of estimated effects between model specifications in the IPTW analysis (see Figure S.7), however the overall conclusions do not depend on which model is used.

Table S.5: Estimates of the effect of insulin use for 1-5 years on FEV_1% obtained using Models 1-3.

Method	Year	Model 1	Model 2	Model 3
IPTW	1	1.25 (0.05, 2.44)	-0.39 (-1.12, 0.35)	-0.61 (-1.16, -0.06)
	2	-0.50 (-1.79, 0.79)	0.80 (-0.29, 1.89)	-0.20 (-1.08, 0.68)
	3	-1.11 (-2.75, 0.53)	0.09 (-1.30, 1.48)	-0.07 (-1.31, 1.18)
	4	-3.23 (-5.74, -0.71)	-0.99 (-2.81, 0.83)	-0.09 (-1.72, 1.53)
	5	-0.55 (-3.64, 2.55)	0.28 (-2.24, 2.80)	0.45 (-2.09, 2.98)
G-formula	1	0.22 (-0.38, 0.82)	0.23 (-0.45, 0.91)	0.22 (-0.87, 1.31)
	2	-0.59 (-1.43, 0.26)	-0.46 (-1.78, 0.87)	0.05 (-1.38, 1.48)
	3	-1.37 (-2.53, -0.21)	-1.14 (-2.87, 0.59)	-0.88 (-2.67, 0.91)
	4	-1.77 (-3.18, -0.36)	-1.81 (-4.02, 0.39)	-1.90 (-4.29, 0.49)
	5	-1.75 (-3.48, -0.02)	-1.00 (-3.79, 1.78)	-1.40 (-4.23, 1.43)

Figure S. 6: Estimates of the effect of insulin use for 1-5 years on FEV1% obtained using Models 1-3

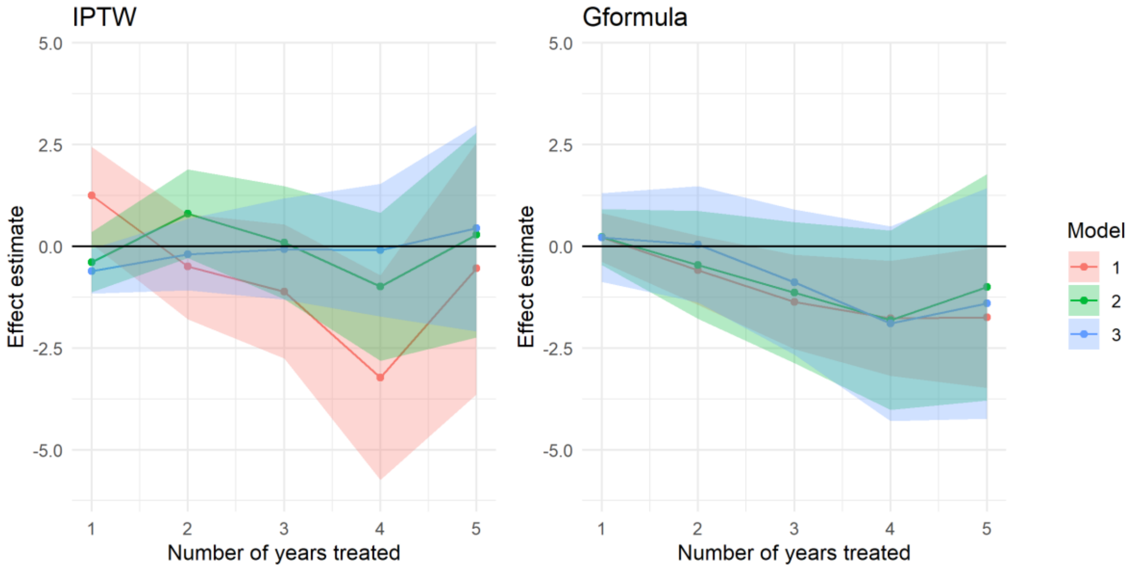

4.2.4 Sensitivity to assumptions about causal pathways

Table S.6 and Figure S.8 show the estimated effects of insulin use for 1-5 years on FEV_1% obtained in the original analysis (using G-formula and Model 1, defined in Section 1.3) and a sensitivity analysis. This analysis assessed the sensitivity of our findings to violations of the DAG assumptions. The tabulated values and figure shows that our results are not sensitive to changes in assumptions about the direction of causal pathways in the DAG.

Table S.6: Estimates of the effect of insulin use for 1-5 years on FEV_1% in the original analysis and a sensitivity to the direction of causal pathways analysis

Year	Original Analysis	Sensitivity analysis
1	0.22 (-0.38, 0.82)	0.22 (-0.40, 0.82)
2	-0.59 (-1.43, 0.26)	-0.59 (-1.43, 0.26)
3	-1.37 (-2.53, -0.21)	-1.37 (-2.53, -0.21)
4	-1.77 (-3.18, -0.36)	-1.77 (-4.02, -0.36)
5	-1.75 (-3.48, -0.02)	-1.75 (-3.48, -0.02)

Figure S. 7: Estimates of the effect of insulin use for 1-5 years on FEV1% in the original analysis and a sensitivity to the direction of causal pathways analysis

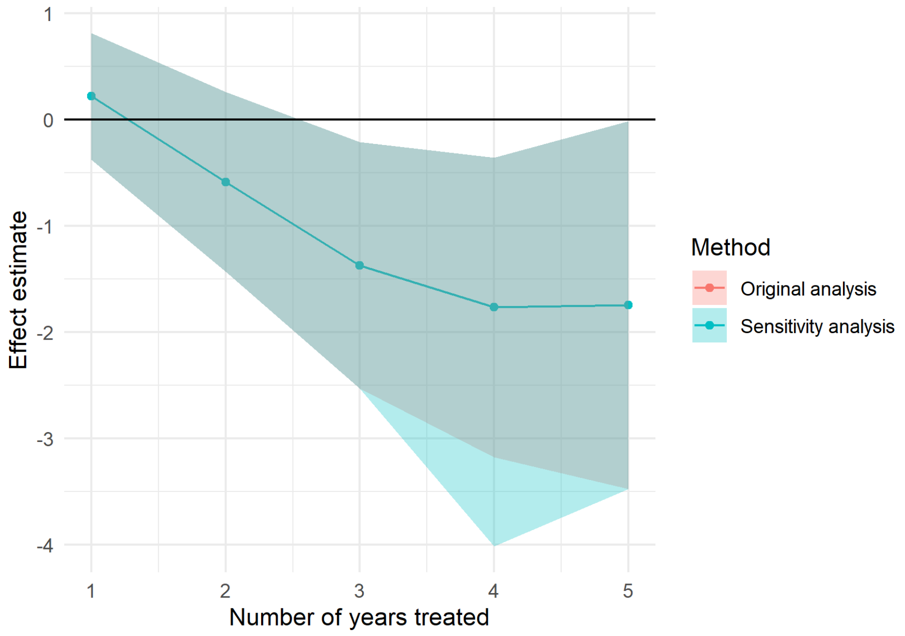

4.2.5 Including rate of decline in FEV_1% as a time-varying confounder

Table S.7 and Figure S.9 show the estimated effects of insulin use for 1-5 years on FEV_1% obtained in the original analysis (using IPTW and Model 1, defined in Section 1.3) and a sensitivity analysis. This analysis assessed how including the rate of decline in FEV_1% as a time-varying confounder affected the results. Results indicate that including the rate of decline in FEV_1% as a time-varying confounder had very little impact on the results.

Table S.7: Estimates of the effect of insulin use for 1-5 years on FEV_1% in the original analysis and a sensitivity analysis which includes rate of decline in FEV_1% as a time-varying confounder

Year	Original Analysis	Sensitivity analysis
1	1.25 (0.05, 2.44)	1.20 (-0.01, 2.40)
2	-0.50 (-1.79, 0.79)	-0.63 (-1.92, 0.67)
3	-1.11 (-2.75, 0.53)	-1.15 (-2.79, 0.50)
4	-3.23 (-5.74, -0.71)	-3.23 (-5.76, -0.70)
5	-0.55 (-3.64, 2.55)	-0.50 (-3.56, 2.56)

Figure S. 8: Estimates of the effect of insulin use for 1-5 years on FEV1% in the original analysis and a sensitivity analysis which includes rate of decline in FEV_1% as a time-varying confounder

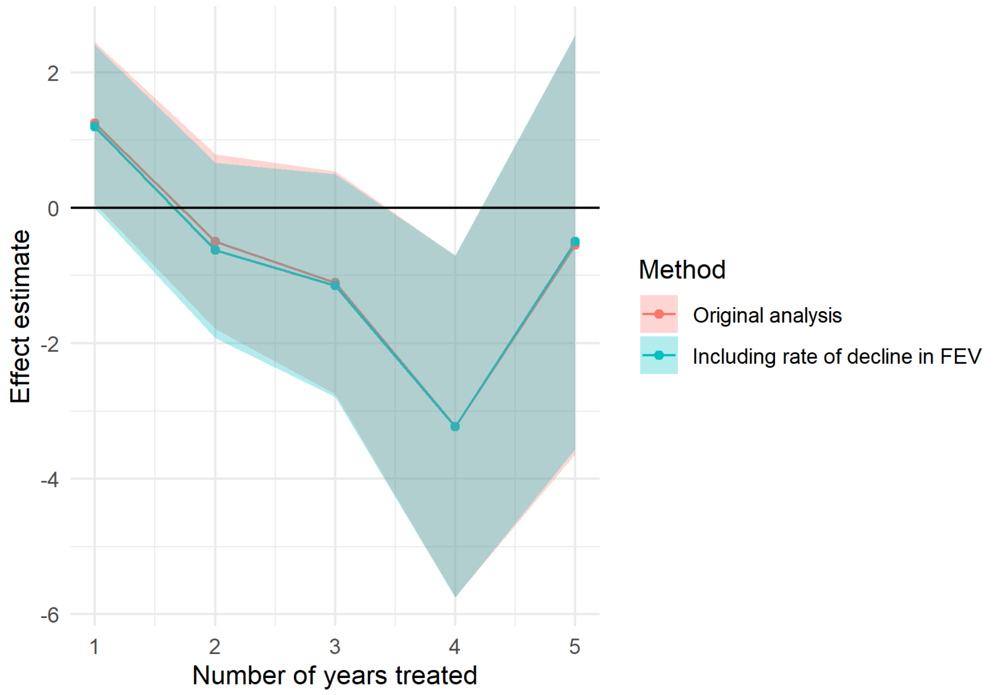

4.2.6 Unadjusted analyses

Table S.8 and Figure S.10 compare the estimated effects of insulin use for 1-5 years on FEV_1% obtained in the original analysis, to partially (partial: only baseline confounders were adjusted for) and completely (none: no confounders were adjusted for) unadjusted analyses. For IPTW, the partially and completely unadjusted results are very similar to the original analysis after year 1, however, for the G-formula, the unadjusted analyses do differ from the original analysis. The partial analysis provided no evidence of an effect overall and the results in the completely unadjusted analysis indicate that the treatment effect improves over time (this is the opposite to what was observed in the original analysis). For both IPTW and the G-formula, the confidence intervals were consistently wider for the completely unadjusted analysis.

Table S.8: Estimates of the effect of insulin use for 1-5 years on FEV_1% in three analyses: original analysis, partial (time-invariant confounders were adjusted for, but not time-varying confounders) and none (neither time-varying nor time-invariant confounders were adjusted for)

Method	Year	Original Analysis	Partial	None
IPTW	1	1.25 (0.05, 2.44)	0.94 (-2.03, 3.91)	0.17 (-2.80, 3.14)
	2	-0.50 (-1.79, 0.79)	-0.60 (-3.65, 2.44)	0.10 (-2.94, 3.14)
	3	-1.11 (-2.75, 0.53)	-2.01 (-5.54, 1.52)	-1.34 (-4.89, 2.17)
	4	-3.23 (-5.74, -0.71)	-3.83 (-7.98, 0.32)	-2.99 (-7.14, 1.16)
	5	-0.55 (-3.64, 2.55)	-2.86 (-7.69, 1.98)	0.29 (-4.55, 5.13)
G-formula	1	0.22 (-0.38, 0.82)	0.25 (-0.35, 0.85)	-4.28 (-7.82, -0.75)
	2	-0.59 (-1.43, 0.26)	0.19 (-0.85, 1.22)	-3.55 (-7.04, -0.07)
	3	-1.37 (-2.53, -0.21)	0.17 (-1.00, 1.34)	-3.47 (-6.89, -0.06)
	4	-1.77 (-3.18, -0.36)	-0.18 (-1.37, 1.02)	-3.10 (-6.37, 0.16)
	5	-1.75 (-3.48, -0.02)	-0.58 (-1.81, 0.65)	-3.36 (-6.53, -0.20)

Figure S. 9: Estimates of the effect of insulin use for 1-5 years on FEV1% in three analyses: original analysis, partial (time-invariant confounders were adjusted for, but not time-varying confounders) and none (neither time-varying nor time-invariant confounders were adjusted for)

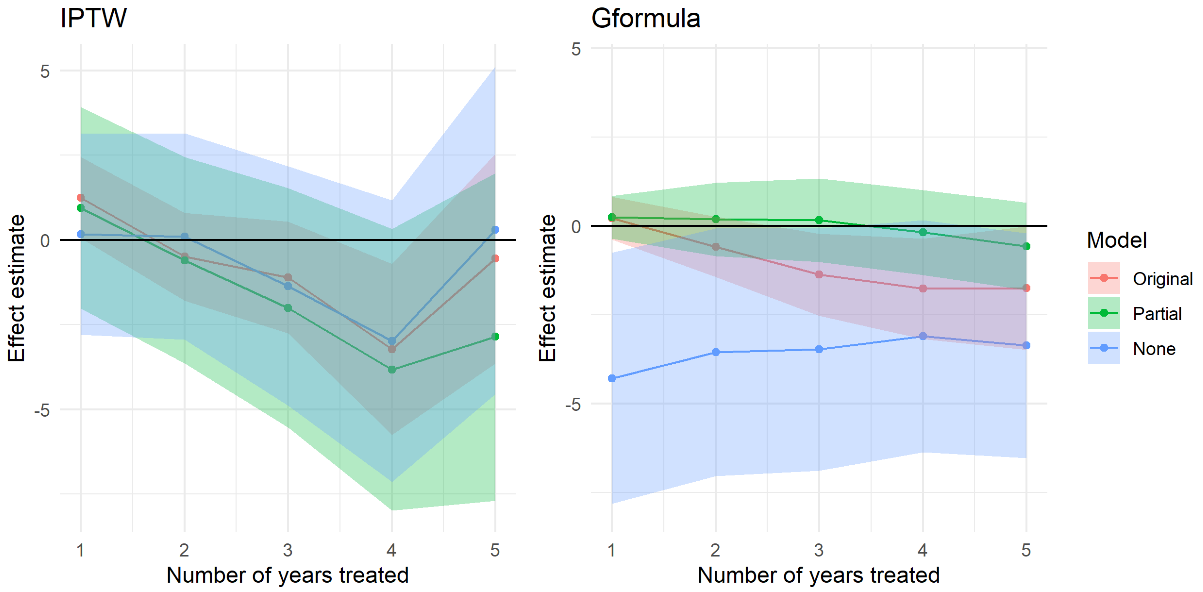

4.3 BMI z-score as outcome

4.3.1 Distribution of weights

Figures S.11 and S.12 show the distribution of inverse-probability-of-treatment weights and overall weights by year, respectively. Boxplots show that the median weights are approximately 1 for each year, as expected. The variance of weights increases by year but there are no extreme values.

Figure S. 10: Distribution of inverse-probability-of-treatment weights by year

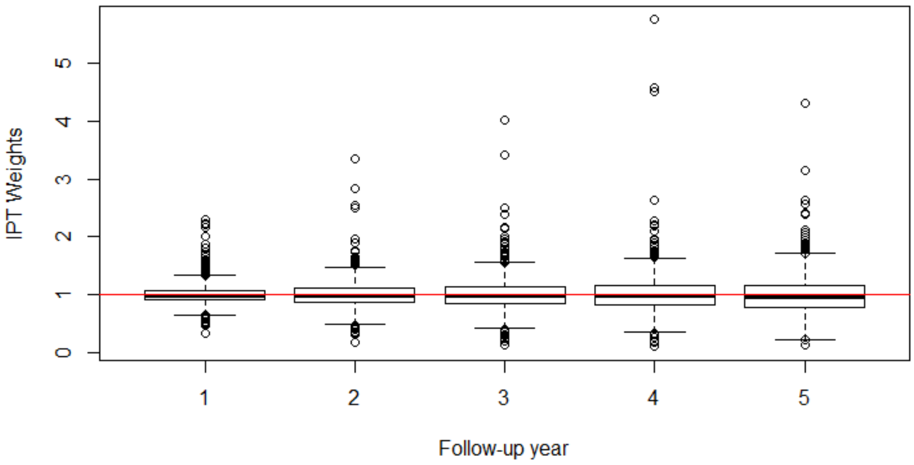

Figure S. 11: Distribution of overall weights by year

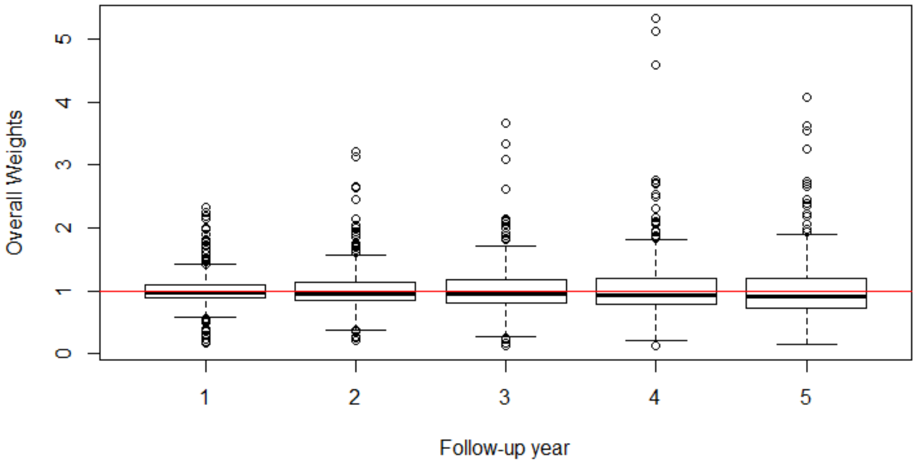

4.3.2 Tabulated values for the models with an interaction term

Table S.9 shows the estimated effects of insulin use for 1-5 years on BMI z-score for the whole cohort, and conditional on high, moderate or low BMI z-score at baseline. Estimated effects for the whole cohort are obtained using Model 1 and conditional estimates are obtained using Model 1b, as defined in Section 1.3. For both the IPTW and G-formula results, estimated effects tend to increase as baseline BMI decreases.

Table S.9: Estimates of the effect of insulin use for 1-5 years on BMI z-score for the whole cohort, and conditional on high, moderate or low BMI at baseline, obtained using inverse-probability-of-weighting (IPTW) and the G-Formula

Year	BMI z-score	IPTW	G-formula
1	Whole cohort	0.02 (-0.06, 0.09)	0.03 (-0.01, 0.07)
	80	-0.03 (-0.14, 0.08)	-0.01 (-0.06, 0.05)
	50	0.01 (-0.06, 0.08)	0.02 (-0.01, 0.06)
	20	0.06 (-0.07, 0.19)	0.06 (-0.01, 0.13)
2	Whole cohort	0.03 (-0.08, 0.14)	0.03 (-0.03, 0.10)
	80	-0.02 (-0.15, 0.12)	-0.06 (-0.13, 0.01)
	50	0.03 (-0.08, 0.13)	0.01 (-0.06, 0.07)
	20	0.08 (-0.08, 0.23)	0.08 (-0.02, 0.18)
3	Whole cohort	-0.01 (-0.14, 0.13)	0.04 (-0.04, 0.12)
	80	-0.05 (-0.20, 0.10)	-0.03 (-0.11, 0.05)
	50	-0.06 (-0.14, 0.13)	-0.01 (-0.09, 0.07)
	20	0.04 (-0.14, 0.22)	0.02 (-0.11, 0.15)
4	Whole cohort	-0.15 (-0.32, 0.03)	-0.00 (-0.10, 0.10)
	80	-0.19 (-0.37, -0.01)	-0.17 (-0.27, -0.06)
	50	-0.14 (-0.32, 0.03)	-0.09 (-0.20, 0.01)
	20	-0.10 (-0.31, 0.12)	-0.01 (-0.19, 0.17)
5	Whole cohort	-0.10 (-0.33, 0.14)	0.00 (-0.13, 0.13)
	80	-0.14 (-0.38, 0.10)	-0.13 (-0.30, 0.03)
	50	-0.10 (-0.34, 0.14)	-0.10 (-0.22, 0.08)
	20	-0.05 (-0.32, 0.22)	0.01 (-0.23, 0.25)

4.3.3 Sensitivity to model specification

Table S.10 and Figure S.13 show the estimated effects of insulin use for 1-5 years on BMI z-score obtained using Models 1-3 (defined in Section 1.5). Results indicate that the overall conclusions are not sensitive to model specification.

Table S.10: Estimates of the effect of insulin use for 1-5 years on BMI z-score obtained using Models 1-3.

Method	Year	Model 1	Model 2	Model 3
IPTW	1	0.02 (-0.06, 0.09)	0.03 (-0.02, 0.09)	-0.01 (-0.05, 0.04)
	2	0.03 (-0.08, 0.14)	0.05 (-0.03, 0.12)	0.00 (-0.07, 0.08)
	3	-0.01 (-0.14, 0.13)	0.05 (-0.06, 0.17)	0.01 (-0.09, 0.11)
	4	-0.15 (-0.32, 0.03)	-0.02 (-0.16, 0.11)	-0.02 (-0.16, 0.12)
	5	-0.10 (-0.33, 0.14)	-0.11 (-0.3, 0.08)	-0.06 (-0.25, 0.12)
G-formula	1	0.03 (-0.01, 0.07)	0.00 (-0.04, 0.04)	0.01 (-0.05, 0.08)
	2	0.03 (-0.03, 0.10)	0.15 (0.04, 0.26)	0.14 (0.03, 0.25)
	3	0.04 (-0.04, 0.12)	0.08 (-0.05, 0.21)	0.10 (-0.03, 0.23)
	4	-0.00 (-0.10, 0.10)	-0.06 (-0.23, 0.10)	-0.07 (-0.24, 0.09)
	5	0.00 (-0.13, 0.13)	0.03 (-0.17, 0.23)	0.04 (-0.15, 0.23)

Figure S. 12: Estimates of the effect of insulin use for 1-5 years on BMI z-score obtained using Models 1-3

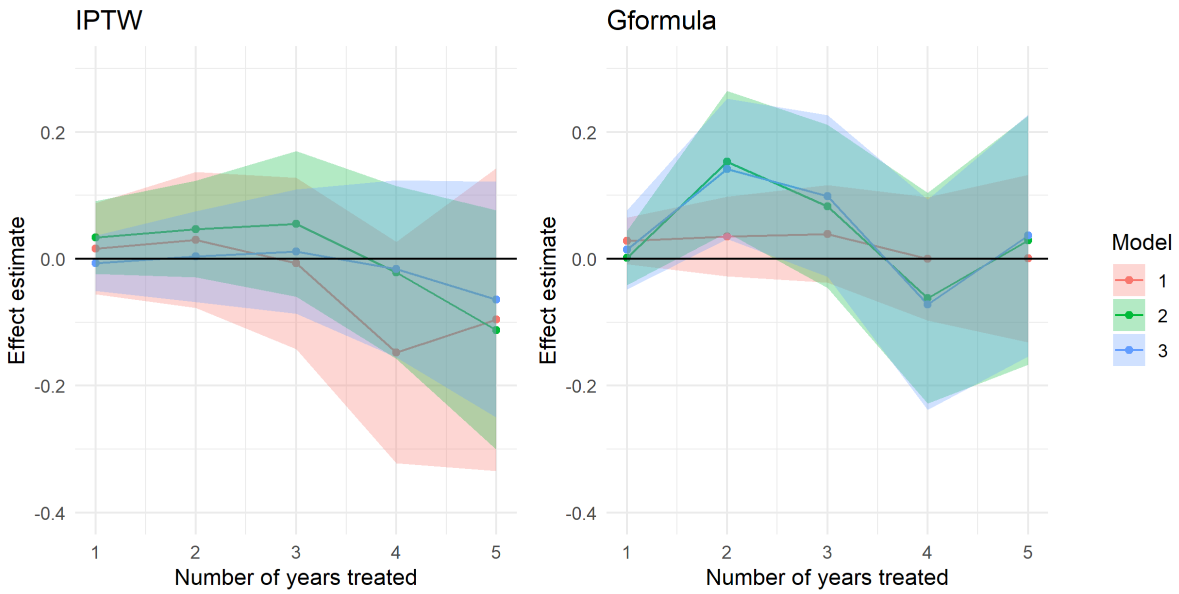

4.3.4 Sensitivity to causal pathways

Table S.11 and Figure S.14 show the estimated effects of insulin use for 1-5 years on BMI z-score obtained in the original analysis (using G-formula and Model 1, defined in Section 1.3) and a sensitivity analysis. This analysis assessed the sensitivity of our findings to violations of the DAG assumptions. The tabulated values and figure shows that our results are not sensitive to changes in assumptions about the direction of causal pathways in the DAG.

Table S.11: Estimates of the effect of insulin use for 1-5 years on BMI z-score in the original analysis and a sensitivity to the direction of causal pathways analysis

Year	Original Analysis	Sensitivity analysis
1	0.03 (-0.01, 0.07)	0.02 (-0.01, 0.07)
2	0.03 (-0.03, 0.10)	0.06 (-0.03, 0.10)
3	0.04 (-0.04, 0.12)	0.04 (-0.04, 0.12)
4	-0.00 (-0.10, 0.10)	0.01 (-0.10, 0.10)
5	0.00 (-0.13, 0.13)	0.03 (-0.13, 0.13)

Figure S. 13: Estimates of the effect of insulin use for 1-5 years on BMI z-score in the original analysis and a sensitivity to the direction of causal pathways analysis

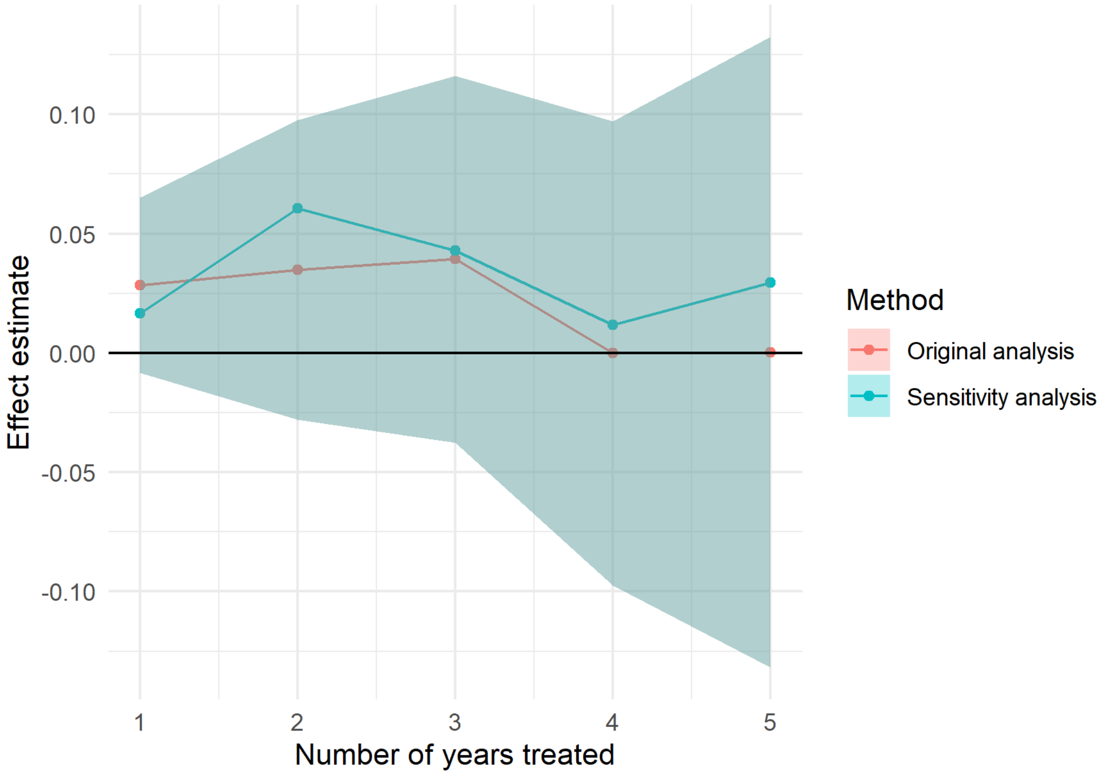

4.3.5 Including rate of decline in FEV_1% as a time-varying confounder

Table S.12 and Figure S.15 show the estimated effects of insulin use for 1-5 years on BMI z-score obtained in the original analysis (using IPTW and Model 1, defined in Section 1.3) and a sensitivity analysis. There are differences in the results between the original analysis and sensitivity analysis presented here. However, similarly to the equivalent sensitivity analysis with FEV_1% as the outcome, including the rate of decline in FEV_1% as a time-varying confounder had negligible effect on the results.

Table S.12: Estimates of the effect of insulin use for 1-5 years on BMI z-score in the original analysis and a sensitivity analysis which includes rate of decline in FEV_1% as a time-varying confounder

Year	Original Analysis	Sensitivity analysis
1	0.02 (-0.06, 0.09)	0.01 (-0.06, 0.08)
2	0.03 (-0.08, 0.14)	0.03 (-0.08, 0.14)
3	-0.01 (-0.14, 0.13)	-0.01 (-0.15, 0.12)
4	-0.15 (-0.32, 0.03)	-0.15 (-0.32, 0.03)
5	-0.10 (-0.33, 0.14)	-0.08 (-0.32, 0.16)

Figure S. 14: Estimates of the effect of insulin use for 1-5 years on BMI z-score in the original analysis and a sensitivity analysis which includes rate of decline in FEV1% as a time-varying confounder

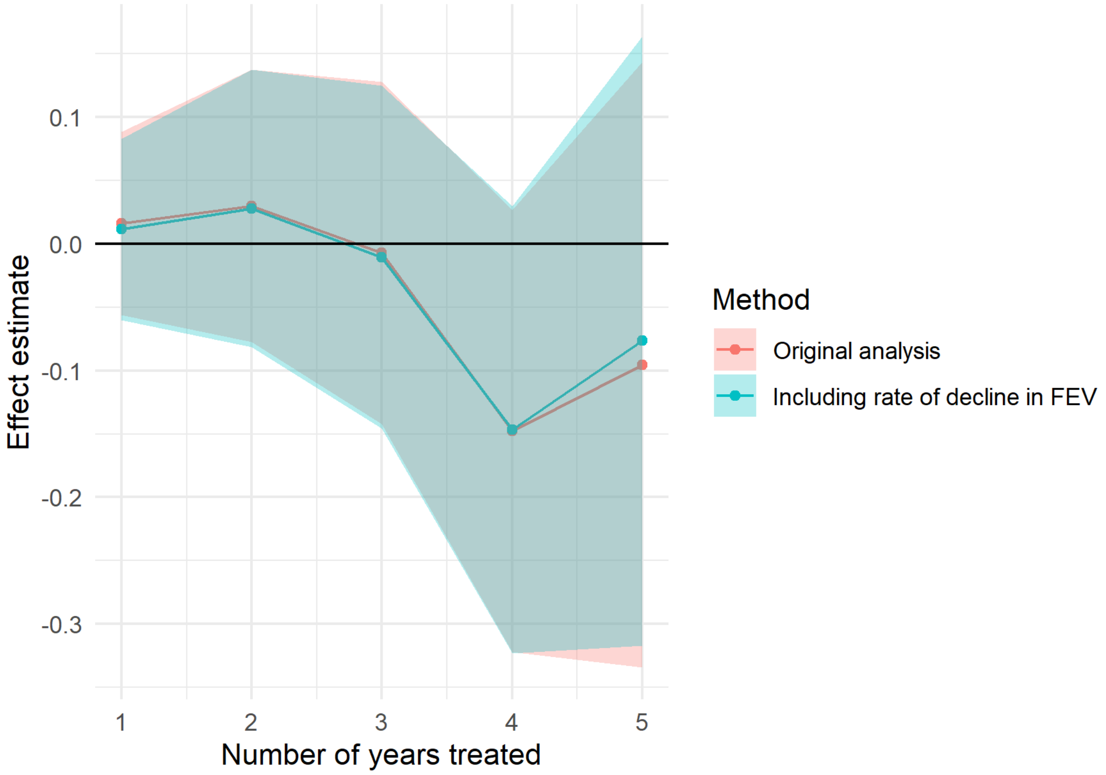

4.3.6 Unadjusted analyses

Table S.13 and Figure S.16 compare the estimated effects of insulin use for 1-5 years on BMI z-score obtained in the original analysis, to partially (partial: only baseline confounders were adjusted for) and completely (none: no confounders were adjusted for) unadjusted analyses. For both IPTW and the G-formula, the completely unadjusted analysis gave negative treatment effects at all time points, when are then attenuated after partial adjustment. The results from the partial adjustment are similar to those in the original analysis, indicating that most of the confounding we have controlled for was baseline confounding.

Table S.13: Estimates of the effect of insulin use for 1-5 years on BMI z-score in three analyses: original analysis, partial (time-invariant confounders were adjusted for, but not time-varying confounders) and none (neither time-varying nor time-invariant confounders were adjusted for)

Method	Year	Original Analysis	Partial	None
IPTW	1	0.02 (-0.06, 0.09)	0.02 (-0.05, 0.09)	-0.16 (-0.34, 0.02)
	2	0.03 (-0.08, 0.14)	0.07 (-0.04, 0.17)	-0.08 (-0.28, 0.13)
	3	-0.01 (-0.14, 0.13)	-0.03 (-0.15, 0.09)	-0.14 (-0.37, 0.08)
	4	-0.15 (-0.32, 0.03)	-0.20 (-0.36, -0.03)	-0.39 (-0.65, -0.13)
	5	-0.10 (-0.33, 0.14)	-0.17 (-0.27, 0.026)	-0.15 (-0.47, 0.17)
G-formula	1	0.03 (-0.01, 0.07)	0.02 (-0.02, 0.05)	-0.14 (-0.33, 0.06)
	2	0.03 (-0.03, 0.10)	0.06 (-0.01, 0.12)	-0.15 (-0.34, 0.05)
	3	0.04 (-0.04, 0.12)	0.10 (0.02, 0.18)	-0.06 (-0.26, 0.13)
	4	-0.00 (-0.10, 0.10)	0.09 (0.01, 0.17)	-0.08 (-0.27, 0.11)

5	0.00 (-0.13, 0.13)	0.08 (-0.01, 0.16)	-0.07 (-0.25, 0.11)

Figure S. 15: Estimates of the effect of insulin use for 1-5 years on BMI z-score in three analyses: original analysis, partial (time-invariant confounders were adjusted for, but not time-varying confounders) and none (neither time-varying nor time-invariant confounders were adjusted for).

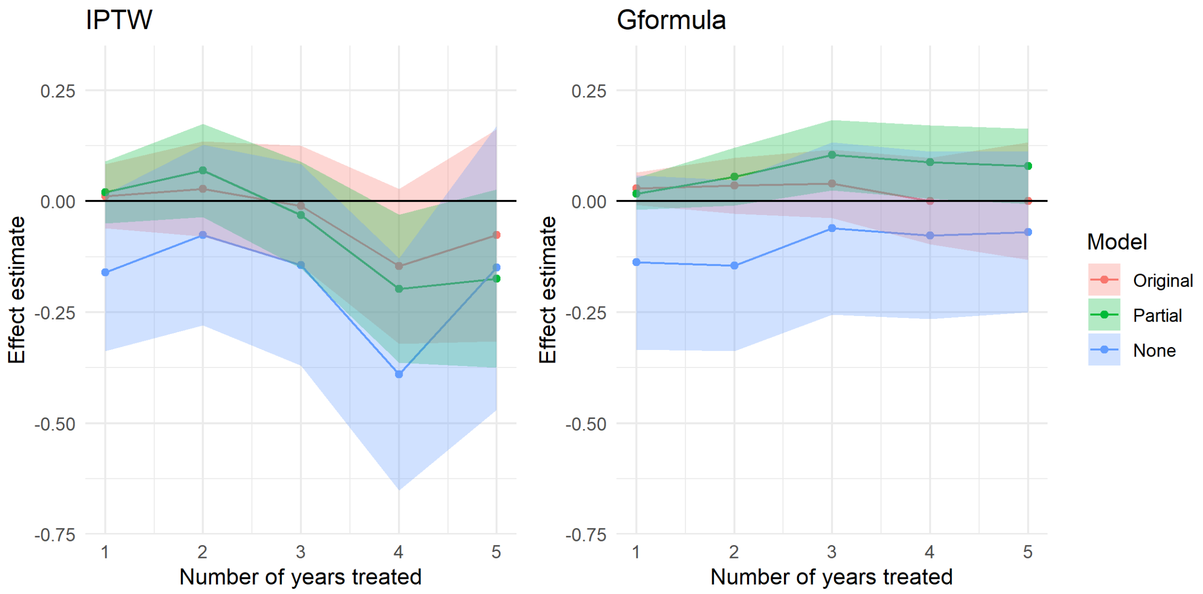

Supplement: Supplementary file 1 [file 00170-2022.SUPPLEMENT.pdf]
